# Engineering and Expression Strategies for Optimization of L-Asparaginase Development and Production

**DOI:** 10.3390/ijms242015220

**Published:** 2023-10-16

**Authors:** Anastasiya N. Shishparenok, Yulia A. Gladilina, Dmitry D. Zhdanov

**Affiliations:** 1 Laboratory of Medical Biotechnology, Institute of Biomedical Chemistry, Pogodinskaya St. 10/8, 119121 Moscow, Russia; a.shishparyonok@yandex.ru (A.N.S.); leonova_y@mail.ru (Y.A.G.); 2 Department of Biochemistry, Peoples’ Friendship University of Russia named after Patrice Lumumba (RUDN University), Miklukho—Maklaya St. 6, 117198 Moscow, Russia

**Keywords:** L-asparaginase, directed evolution, rational design, heterologous expression, genetic engineering, computer design of enzyme

## Abstract

Genetic engineering for heterologous expression has advanced in recent years. Model systems such as *Escherichia coli*, *Bacillus subtilis* and *Pichia pastoris* are often used as host microorganisms for the enzymatic production of L-asparaginase, an enzyme widely used in the clinic for the treatment of leukemia and in bakeries for the reduction of acrylamide. Newly developed recombinant L-asparaginase (L-ASNase) may have a low affinity for asparagine, reduced catalytic activity, low stability, and increased glutaminase activity or immunogenicity. Some successful commercial preparations of L-ASNase are now available. Therefore, obtaining novel L-ASNases with improved properties suitable for food or clinical applications remains a challenge. The combination of rational design and/or directed evolution and heterologous expression has been used to create enzymes with desired characteristics. Computer design, combined with other methods, could make it possible to generate mutant libraries of novel L-ASNases without costly and time-consuming efforts. In this review, we summarize the strategies and approaches for obtaining and developing L-ASNase with improved properties.

## 1. Introduction

L-asparaginase (L-ASNase, E.C.3.5.1.1) hydrolyzes asparagine to produce ammonia and L-aspartic acid. The enzyme is widely used in the pharmaceutical and food industries. L-ASNase is a key therapeutic component in the treatment of lymphosarcoma and acute lymphoblastic leukemia (ALL) [1,2,3,4,5]. Recent research has shown that L-ASNase has clinical potential in the treatment of several aggressive subtypes of hematological or solid tumors, including glioblastoma, breast, pancreatic and hepatocellular carcinomas [6,7]. The anticancer effect of L-ASNase is based on its ability to hydrolyze L-asparagine, which is necessary for neoplastic cells. Leukemic cells cannot synthesize this amino acid due to the absence or lack of L-asparagine synthetase and depend on the exogenous supply from the bloodstream. Depletion of asparagine leads to impaired protein synthesis and starvation of cancer cells, leading to cell death [1]. The second view, or non-canonical approach, is that L-ASNase acts directly on cancer cells. The effects of ASNase on ROS levels, cell cycle progression, autophagy and apoptotic cell death have been demonstrated. Another anti-cancer strategy under L-ASNase treatment is the inhibition of the Akt/mTOR and Erk pathways [8]. L-ASNase is also a promising agent for the food industry because it reduces the formation of toxic acrylamide. Acrylamide is formed as a result of the nonenzymatic interaction of sugars with L-asparagine when starchy foods are heated to 120 °C under low humidity conditions in the Maillard reaction [9,10,11]. Effects unrelated to the hydrolysis of asparagine or glutamine have been described for a number of L-ASNases [12,13,14].

The pharmaceutical and food industries require high productivity, ease of handling and scaling-up, stability (temperature, pH, storage), high enzymatic activity, low toxicity, simplicity of product purification and low production costs before designing and scaling up bioprocesses [15]. Many commercial enzymes now use a combination of protein engineering and rational design methods based on computer modeling of proteins, which complements protein engineering and makes it more precise and efficient [16]. L-ASNases for therapeutic use are currently produced exclusively from *Escherichia coli* and *Erwinia chrysanthemi* [17], which are FDA-approved drugs [8]. Currently, the World Health Organization (WHO) has included L-ASNase in the list of essential medicines [18]. It is marketed under several brand names, including Kidrolase^®^, Elspar^®^, Leunase^®^ and Spectrila^®^. However, both standard and pegylated L-ASNase are usually given in combination with other anti-cancer drugs such as vincristine, mercaptopurine, methotrexate, daunorubicin and prednisone [19].

Fungal L-ASNases derived from *Aspergillus niger* and *A. oryzae* have also been approved for use in the food sector [20]. Commercially available products for the reduction of acrylamide in the food industry are PreventASe^®^ (DSM) and Acrylaway^®^ (Novozymes) [21].

In recent years, there has been increasing interest in investigating the potential of this enzyme as an antibacterial agent. The antimicrobial activity of L-ASNase has been described against a wide range of gram-positive/gram-negative bacteria: *Klebsiella pneumoniae*, *Listeria monocytogenes*, *Proteus vulgaris*, *Pseudomonas aeruginosa*, *Staphylococcus aureus*, *Salmonella typhimurium* [22].

The main task of protein engineering is to optimize the stability and activity of proteins to overcome their natural limitations in harsh environments. Currently, three different approaches are used for enzyme bioengineering: semi-rational, directed evolution, and rational design, depending on the level of information available about the sequence, structure and function of the target enzyme [23]. To improve the various properties of L-ASNases, researchers have used rational design and other molecular approaches [16,24,25,26,27,28,29,30,31,32,33,34,35,36,37]. Rational design methods have been useful in predicting and modifying enzyme structures. These techniques have overcome laboratory-scale problems of high immunogenicity, poor in vivo stability, anti-asparaginase antibody formation and poor thermal stability [20].

The main expression and engineering strategies to obtain and develop improved L-ASNases are described in this review. We have focused on host system engineering, directed evolution and rational design strategies to develop novel L-ASNases with improved properties.

## 2. Host Systems for Expression of L-Asparaginase

L-ASNases are widespread in nature [38]. According to the National Centre for Biotechnology Information (NCBI), L-ASNase sequences are mostly found in bacteria, accounting for 95.5% of all protein sequences deposited (221,303 out of 231,770). However, L-ASNAse can also be found in the fungi (1.68%), animal (1.25%), plant (0.24%), archaea (0.88%) and kingdoms [39]. 

The best producers of L-ASNase belong to the family Enterobacteriaceae, followed by species of fungi. The main hosts for L-ASNase expression systems to date are *E. coli*, *Bacillus subtilis* (*B. subtilis*) and *Pichia pastoris* (*P. pastoris*) [40]. Among bacteria, wild types of Bacillus are natural producers of L-ASNase, e.g., *Bacillus australimaris* NJB19 (MG734654) [41] and *Bacillus lichenformis* [42].

L-ASNases have been discovered producing enzymes in eukaryotes, such as fungi and yeasts, that have fewer negative consequences and greater beneficial properties [43]. Eukaryotic systems offer several advantages such as proper folding, efficient secretion, and typical eukaryotic post-translational modifications [43,44,45]. A number of yeast genera with considerable L-ASNase production capacity have been documented, mainly from the genera Candida, Yarrowia and Rhodotorula, whereas Aspergillus, Penicillium and Fusarium dominate the literature for L-ASNase-producing mold genera [46,47]. Although fungal L-ASNases are often produced extracellularly, which facilitates downstream purification [48], *S. cerevisiae* strains were found to produce both intracellular and extracellular forms of L-ASNase. Blue-green microalgae is an alternative source of L-ASNase that is attractive due to its lack of seasonal variation, low cost of media formulation, and ease of growth and harvest [49]. 

Human cells were also used to express a recombinant bacterial L-ASNase. HEK293 showed highly comparable relative activity profiles with L-ASNase produced in *E. coli* at different pH conditions and temperatures [50]. Recombinant human L-ASNase (ASRGL1) was obtained in Baculovirus and mammalian cells and can also be purchased from the MyBioSource company [51]. 

However, microorganisms such as bacteria, filamentous fungi and yeast are considered to be the best source of L-ASNase due to their ability to grow rapidly on extremely simple and inexpensive substrates. The production of L-ASNase can also be easily genetically modified, depending on the strain used, making the extraction and purification process commercially viable [43]. Additionally, the biotechnological production process is usually easier to optimize and scale-up than other processes [46]. Examples of heterologous expression of L-ASNases in various hosts are described in detail by Castro et al. [15].

L-ASNases are currently classified into three major groups: bacterial type L-ASNase (Class 1, containing classification Types I and II), plant type L-ASNase (Class 2, Type III), and rhizobial type L-ASNase (Class 3) [15,52] Cytosolic bacterial type I L-ASNases are involved in nitrogen metabolism and appear to be expressed constitutively [53]. In mesophiles, these enzymes exhibit low affinity towards L-asparagine with KM values in the millimolar range. Periplasmic bacterial type II L-ASNases appear to participate in carbon metabolism and their expression is tightly regulated by different factors [54]. Type II enzymes of mesophilic origin exhibit a high specific activity towards L-asparagine with micromolar K_M_. Plant-type L-ASNases have dual L-asparaginase and isoaspartyl aminopeptidase activity. These enzymes are located in the periplasmic space and hydrolyze the side-chain amide bond of L-asparagine or its β-peptides [54]. They are low-affinity proteins (millimolar K_M_) that belong to the superfamily of N-terminal nucleophile hydrolases [55,56]. 

Based on structural and kinetic data, two general catalytic mechanisms have been proposed for Class 1 L-ASNases (Type I, Type II): a single displacement (or direct displacement) mechanism for mammalian enzymes and a double displacement (ping-pong) mechanism for bacterial enzymes [57], which is thought to proceed via a covalent acyl-enzyme intermediate. In Class 1 enzymes (bacterial type), the N-terminal domain has all the elements necessary for catalysis [53]. Type II L-ASNases have a molecular weight that varies between 31.0 and 42.0 kDa [58]. Most marine L-ASNases from different sources have molecular masses in the range of 25–41.1 kDa, with the exception of *Bacillus pumilus* L-ASNase, which is active as a dimer or tetramer with a molecular mass of 71 kDa [52]. L-ASNases of the plant type (Type III, Class 2) are found in microorganisms, insects and mammals, including humans. They are Ntn hydrolases with dual isoaspartyl aminopeptidase/l-asparaginase activity (EC 3.5.1.1/3.4.19.5). Human HsAIII and *E. coli* EcAIII are the most widely studied class 2 L-ASNases [53].

L-ASNases of different classes have different architectures and oligomeric states. EcAI and EcAII, for example, are often homotetramers (dimers of two intimate dimers). Some type I enzymes (e.g., from *Pyrococcus horikoshii*) have been reported to function as (intimate) dimers or hexamers (e.g., from *Thermus thermophilus*). Class 2 enzymes have a completely different architecture—the immature precursor of EcAIII is a homodimer with a sandwich-like shape and mature chains, with each subunit connected by a linker. During autocleavage, a catalytic threonine is released at the N-terminus of the subunit and the linker region is disordered or even partially destroyed. Class 3 enzymes have a unique architecture that is not connected to the structures of Class 1 or 2, but is more akin to serine-lactamases or penicillin-binding proteins [59].

Despite the considerable diversity in L-ASNase amino acid sequences, the architecture and content of their active site pockets, as well as their tertiary structures, are highly conserved. The larger N-terminal domain is covalently linked to the smaller C-terminal domain by a 20–25 amino acid linker in an L-ASNase protomer [60]. Each monomer consists of approximately 330 amino acid residues, comprising 14 β-strands and 8 α-helices organized into 2 distinct domains: the larger N-terminal domain and the smaller C-terminal domain [61]. 

Some of the structural features of an L-ASNase protomer vary slightly between individual enzymes; nevertheless, the overall topology appears to be very highly conserved. The biological assembly of both type I and II ASNases is a homotetramer [62,63]. The whole assembly is relatively compact. The tetramer can be represented as a dimer consisting of two intimate dimers. The interface between protomers within such a small dimer is larger than the interface between protomers from different dimers. Each dimer forms two complete active sites, raising the question of the role of tetramerization. As there are four active sites at the interface of two subunits forming an intimate dimer, L-ASNase is more accurately characterized as a dimer of dimers with an anticancer molecular mass of around 120 to 160 kDa [64]. The active sites of L-ASNases are located at the interfaces of intimate dimers, with each intimate dimer having two active site pockets generated by amino acids from both subunits. Structural and functional studies have shown that the catalytic triad consisting of three polar amino acids, Thr-Lys-Asp (Thr89, Lys162 and Asp90 in EcA), is required for enzyme activity [65]. Investigation of the structure of L-ASNase with ligand molecules in the active site revealed the formation of an intricate hydrogen network with ligands and the discovery of two new residues important for the catalytic mechanism (Thr12 and Tyr25 in EcA) [54]. These residues are located in a broad loop (amino acids 10–32 in EcA) that acts as a lid for the active site, most likely facilitating substrate binding and thus catalysis [46]. 

In terms of amino acid sequences, all known bacterial type II L-ASNases, including those used in medicine, show significant similarity. Because their amino acid sequences are so similar, their tertiary and quaternary structures are also very similar: they fold as homotetramers with four active sites between the N- and C-terminal domains of two adjacent monomers. The tertiary and quaternary structures of bacterial L-ASNases are also very similar. Each monomer contains 40 β-layers and 8 α-helices arranged in an N-terminal domain and a smaller C-terminal region connected by a linker of about 20 amino acid residues [1,60]. When a monomer folds, the binding cavity is partially formed; when two monomers join, the binding cavity is fully formed, creating a binding site that partially sits at the dimer boundary. A loop, which acts as a ‘lid’, covers the top of the binding site. Near the catalytic site, the N24 lid loop residue of EcA participates in a hydrogen bond network [66].

The main problems associated with L-ASNase produced by prokaryotic microorganisms are the induction of chemoresistance in cancer cells and side effects in the form of hypersensitivity of immune responses [67]. L-ASNase causes a variety of adverse effects and consequences, including liver function abnormalities, pancreatitis, diabetes, congestive heart failure, rashes, pyrexia, cytopenia, neurological disorders, and more [18]. 

In industrial production, L-ASNase can be secreted intracellularly so that cell destruction occurs in several stages. To overcome this problem, a variety of molecular tools and techniques are available for high-level expression of heterologous L-ASNases, including the design of expression plasmids, engineered production hosts, and growth and culture strategies [68]. The use of both naturally occurring hyperproductive strains and those created through genetic engineering, in particular CRISPR/Cas9 systems, protoplast fusion, and response surface methodology, as well as the design of experiments using artificial neural networks and response surfaces, has significantly increased yields [69,70]

### 2.1. Escherichia coli Expression System

L-ASNase I and L-ASNase II are the two different forms of L-ASNases that have been found in *E. coli* [71]. Among these, L-ASNase II was the primary source of commercially used L-ASNase [40]. Asparaginase isozyme II (AnsB) from *E. coli* is a prokaryotic protein that has been studied for the treatment of acute lymphoblastic leukemia (ALL) for more than 50 years. Much research has been devoted to the active expression of AnsB in *E. coli* [72]. The advantages of using *E. coli* as a host are well-known: fast growth kinetics, high cell densities, easily available and inexpensive components of media and easy and rapid transformation of exogenous plasmid DNA [73]. However, the *E. coli* expression system has some drawbacks, including a lack of eukaryotic post-translational modifications, limited solubility, inappropriate protein folding, inclusion body formation, endotoxins and inadequate secretion [74].

Expression vectors useful for the production of L-ASNase include vectors derived from bacterial plasmids, bacteriophage, yeast episome, yeast chromosomal elements, viruses such as baculovirus, papovavirus, vaccinia virus, adenovirus, fowl pox virus, pseudorabies virus and retroviruses, and vectors derived from combinations thereof, such as those derived from plasmid [75].

**Figure 1:**
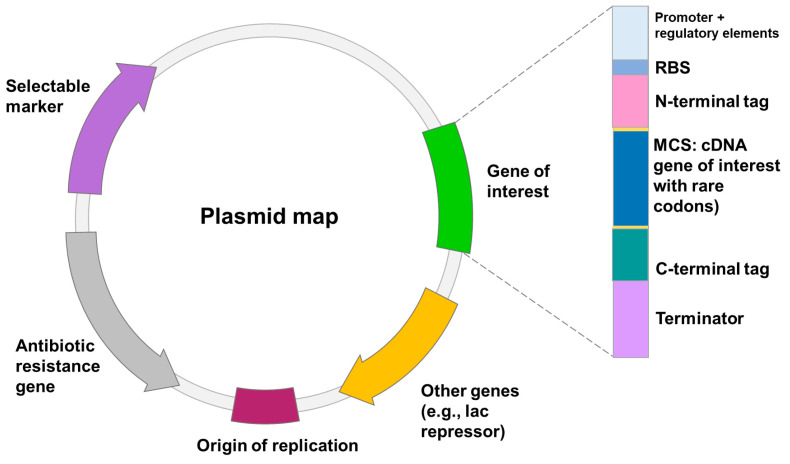
The schematic of the transcription elements on the plasmid to optimize expression of recombinant proteins in *E. coli* and *B. subtilis.* These elements include: (1) origin of replication (ori); (2) translation initiation sequence: a ribosomal-binding site (RBS) and start codon; (3) promoter; (4) affinity tag; (5) multiple cloning site (MCS); (6) transcription termination sequence; (7) selectable marker.

Heterologous gene transcription must be optimized to avoid these shortcomings and to produce competitive biotechnological processes [76]. Today, a wide range of cutting-edge strategies and high-throughput techniques are used to improve recombinant protein expression in *E. coli*. These include strong promoters, novel expression systems, protein tags, codon optimization, secretion signals, *E. coli* engineering for disulfide bond formation and glycoengineering, co-expression of foldases and molecular chaperons, metabolic engineering, high-throughput cloning and screening tools, and fermentation technologies [68]. Elements on the plasmid to optimize heterologous expression are shown in Figure 1.

The most common method to produce recombinant proteins is the use of pET vectors in *E. coli* BL21(DE3) hosts [77]. *E. coli* BL21 (DE3) may also be a favorable host for the production of recombinant proteins, e.g., L-ASNase, because it lacks the cytoplasmic protease Lon and the outer membrane protease OmpT, which can increase the stability of a produced recombinant protein [78,79]. OmpT is best avoided as it is a relatively stable protease that can damage endogenous and recombinant proteins after cell lysis. All of these features combine to make *E. coli* BL21(DE3) a suitable host for recombinant protein production in the cytoplasm and cell envelope, and *E. coli* BL21(DE3) and its variants dominate protein production [80].

**Figure 2:**
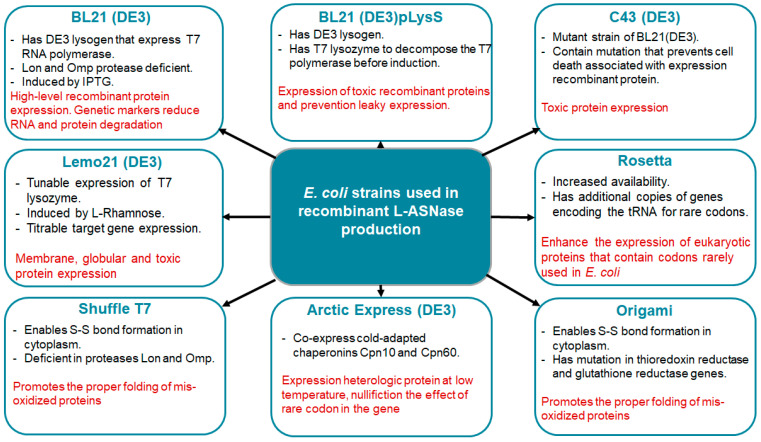
The most popular commercial *E. coli* strains to avoid potential pitfalls of recombinant L-ASNase expression. The key characteristics of the strains are highlighted in black. The advantages are highlighted in red.

Several *E. coli* strains (Figure 2) developed from BL21(DE3) are currently commercially available, each with unique properties to enhance protein expression. The strains represent modifications such as chaperonin co-expression (ArcticExpress(DE3) and GroEL(DE3)), tight expression control (Turner(DE3), pLysS (DE3) and Lemo21(DE3)), cytosolic pro-oxidant environment (Origami B (DE3) and Shuffle T7) and specialization in toxic protein expression (C43(DE3)). Despite the commercial interest in ASNases, only a few strains have been used for enzyme production, the most common being BL21 (DE3). Its protein was produced without a signal peptide for periplasmic export [81]. 

De Moura et al. tested eleven different *E. coli* expression strains to determine the one best suited for the expression of cytosolic L-ASNase at 16 °C using wild-type recombinant EcAII (rEcAII) with a His-tag as the prototype enzyme. BL21 ArcticExpress(DE3) showed the best results among the *E. coli* strains examined, with reduced protein aggregates, correct folding and higher specific activity (156 U/mg), indicating that this strain is perfectly suited for recombinant L-ASNase development [82].

#### 2.1.1. Optimization of Promoter Strength

Promoter engineering is an approach to the development of large numbers of promoter libraries and the regulation of promoters of varying strength and function. Promoter modification is based on mathematical prediction, promoter sequence randomization and promoter hybridization. Although this technique is one of the core areas of metabolic engineering and synthetic biology, it is also useful for the overexpression of recombinant proteins. Three main strategies are used to identify and create new promoters: (1) screening microbial genomes for novel promoters, (2) building large libraries of artificial promoters, and (3) modifying the core region of existing promoters [83].

Promoter strength is one of the key elements influencing metabolic load. Traditionally, promoter engineering efforts have concentrated on creating constitutive and inducible promoters with predictable expression profiles. Designing promoter sequences that accomplish a desired amount of RNA polymerase (RNAP) recruitment to the promoter is the process of tuning the strength of constitutive promoters. Inducible promoters feature locations for transcriptional activators or repressors to bind and adjust transcriptional levels [83,84].

Expression vectors were created using a small number of well-studied gene promoter systems that are still in use today. Promoters used in *E. coli* expression vectors can be divided into three categories depending on their origin and mode of function: (1) *E. coli* native promoters: lac, trp, tac, trc, and ara; (2) viral promoters recognized by *E. coli*: λ_L_, λ_R_, and T5; and (3) T7 and T7_lac_ promoters, which require their own RNAP. The lac promoter and its derivatives (UV5, trc, tac and others) are induced via galactose or IPTG and inhibited by glucose [85]. Promoter araB is induced with L-arabinose and an easily tunable promoter. The viral promoters λ_L_, λ_R_, and T5 are similar to *E.* coli promoters and are recognized by the host itself. The λ_L_ and λ_R_ promoters are hardly used any more, but T5 is found in pQE vectors from Qiagen. T7 promoters are the most commonly used today [86].

BL21 and BL21(DE3) strains are generally preferred for recombinant protein expression. A T7 or T7_lac_ promoter or promoters recognized by *E. coli* RNAP, such as lac, tac, trc, ParaBAD, PrhaBAD and the T5 promoter, are suitable for the expression of a gene in the BL21 (DE3) strain. Since the BL21 strain lacks the gene for T7 RNA polymerase, it can only express proteins from promoters that are recognized by *E. coli* RNA polymerase, such as the lac, tac, trc, ParaBAD, PrhaBAD and T5 promoters [87].

Researchers have created *E. coli* strains (Figure 2) with regulatable promoters for the RNAP gene that can strictly regulate its expression to reduce these effects on the host cell [76]. The BL21(DE3) strain is the gold standard for recombinant protein production. This is primarily due to the prophage T7 RNA polymerase (RNAP) in the BL21(DE3) genome, which can uniquely recognize the T7 promoter (PT7) on the pET plasmid and transcribe at eight times the rate of native *E. coli* RNAP. The most commonly used induction system is the DE3 lysogen/T7 promoter combination. T7 RNAP from the bacterial genome is expressed by the DE3 lysogen under the control of the lac repressor, which is activated by IPTG. T7 RNAP is then available to transcribe the gene of interest from the plasmid T7 promoter. However, T7 RNAP sometimes has a negative effect on recombinant protein levels [85]. In recent years, several BL21(DE3)-derived strains have been widely used to produce different types of recombinant proteins, including C41/C43 (DE3), BL21(DE3)-pLysS, BL21Star(DE3), and SixPack [88]. 

Techniques for optimizing T7 RNAP and pET plasmid expression include (1) optimization of T7 RNAP transcription and translation levels, including promoter substitutions and mutations in the promoter functional region and RBS sequence; (2) regulation of T7 RNAP activity. The traditional method of regulation is the use of lysozyme or light induction; (3) optimization of pET plasmids based on expression intensity and copy numbers.

One of the best ways to adjust T7 RNAP activity is to modify critical amino acid residues. These modifications can either lower the enzyme’s capacity to bind to the T7 promoter or increase the activity of its catalytic function. For instance, the development of derivative hosts, such as BL21(DE3)-pLysE and Lemo21(DE3), based on this idea has allowed for the effective regulation of T7 RNAP activity [88].

Many variant hosts have been produced from BL21(DE3), but increasing protein expression remains a serious challenge in biotechnology. To date, protein expression systems have been extensively optimized using a variety of methods, including host reconstruction, expression vector redesign and optimization of fermentation conditions [89]. It is interesting to note that consensus-based synthetic *E. coli* RNAP promoters developed by multiple sequence alignment perform quite poorly. Instead, the highest levels of expression are driven by a combination of the standard 35 and 10 elements with less definitive downstream sequences and an ideal environment for protein synthesis initiation and elongation [90].

Expression plasmids based on pET have been widely used for the production of L-ASNases. The optimized T7 promoter system produced the highest specific activity and expression level of the recombinant AnsB gene (encoding L-ASNase) compared to the trc, tac and T7 promoter systems [72]. Recombinant *E. coli* BL21(DE3) with the initial plasmid pET was used to express active L-ASNases from psychrophilic fungi *Sclerotinia borealis*, the thermoacidophilic archaea *Acidilobus saccharovorans* and the thermophilic bacteria *Melioribacter roseus* [91,92,93,94,95,96].

Saeed et al. cloned and expressed the *P. furiosus* L-ASNase coding sequence as a 6× His-tag fusion protein in *E. coli* BL21(DE3) pLysS under the control of the T7 promoter of the pET26b (+) vector. The resulting recombinant enzyme showed the highest substrate specificity for asparagine, while no significant specificity was observed for L-glutamine, urea and acrylamide at 10 mM. The K_m_ and Vmax of the purified recombinant enzyme were 1.623 mM and 105 μmol min^−1^ mg^−1^, respectively. The IC_50_ of purified recombinant L-ASNase was 0.8 IU for the human leukemia cell line THP-1 [97].

The gene (YPTB1411) of L-ASNase from *Yersinia pseudotuberculosis* Q66CJ2 (YpA) was cloned and inserted into an *E. coli* BL21 (DE3) expression system. The *Y. pseudotuberculosis* ansB gene was extracted from a pET23a vector (YPTB1411), purified and placed under the control of the arabinose-inducible araBAD promoter on the pBAD24 vector. The resulting recombinant plasmid, termed pBad24/YpA, was used to transform *E. coli* cells. The biochemical properties of YpA were comparable to those of *E. coli* type II L-ASNase. The K_m_ for L-asparagine was 17 ± 0.9 µM, and the glutaminase activity was quite low, more than 15 times lower than the specific activity for L-asparagine. The antiproliferative effect of YpA was also observed in vitro and in vivo using *E. coli* L-ASNase as a reference [98].

The L-ASNase homolog TK1656 from *Thermococcus kodakaraensis* was cloned and inserted into *E. coli* to study its properties. The TK1656 gene (accession number NC_006624) was cloned and inserted into vector pTZ57 R/T. The TK1656 gene was extracted from the resulting plasmid construct pTZ-Tk1656 and cloned and inserted into pET-21. pET-Tk1656 was used to transform *E. coli* BL21-CodonPlus(DE3)-RIL for TK1656 expression. The TK1656 homolog had significant asparaginase activity (2350 U mg^−1^) but no glutaminase activity. The highest L-ASNase activity was observed at 85 °C and pH 9.5. TK1656 has a K_m_ of 5.5 mM and V_max_ values of 3300 μmol min^−1^ mg^−1^ for L-asparagine hydrolysis [99].

Bacterial cells are known to use different sigma factors to control the gene expression network at different stages of growth or in response to changes in the environment and external stimuli. A collection of synthetic promoters was created to mimic the natural regulation of different sigma factors 70, 38, 32 and 24. The host for promoter characterization was *E. coli* BL21(DE3). Promoters are constructed with two or more types of interlocking sigma factor binding sites. Compared to the T7 promoter, the new synthetic promoter P_21285_ produced higher yields of L-ASNase in *E. coli* [100].

The production of recombinant L-ASNase in heterologous expression systems under different promoters is discussed in detail in the review by Lefin et al. [30].

#### 2.1.2. Signal Sequence

There are a number of reports on the use of the pelB leader sequence from *Erwinia carotovora* to achieve efficient periplasmic expression of L-ASNases. When the pelB leader sequence is linked to a protein, it leads the protein to the bacterial periplasm, where the sequence is removed by a signal peptidase [101].

In one study, the asparaginase gene was combined with an efficient pelB leader sequence and an N-terminal 6-fold histidine tag and cloned under the T7lac promoter for extracellular production of recombinant L-ASNase in *E. coli* strain BLR(DE3). A yield of 20,950 U/L of recombinant L-ASNase was produced using an optimized extracellular medium expression approach. Purification of the recombinant protein using Ni-NTA affinity chromatography resulted in an overall yield of 95 mg/L of purified protein with a recovery of 86%, which is approximately eight times higher than previously published results. The authors also demonstrated that the protein was properly folded and present in the culture supernatant in its active tetrameric form [102]. It was also found that the expression of a recombinant L-ASNase coupled to a pelB leader sequence in BLR(DE3) host cells under an inducible T7lac promoter resulted in optimal extracellular secretion. Surprisingly, the C-terminal his-tag inhibited asparaginase secretion, whereas the N-terminal his-tag had no such effect [93]. L-ASNase expression vectors with the pelB leader sequence were used to rationally design L-ASNase mutants with asparaginase activity but no glutaminase activity that will exhibit a greater therapeutic index [103].

An extracellular L-ASNase expression system was created by substituting the pelB leader sequence for the original ansB gene signal. This modification and optimization of the medium increased the expression level of the enzyme to 130 U/mL [104].

Kim et al. created fusion tag systems in *E. coli* containing the pelB signal sequence and repetitive aspartate tags of varying lengths to promote high expression and extracellular release of the active asparaginase II isoenzyme. With a specific activity of 34.6 U/mg, recombinant LASNase II linked to the pelB signal sequence and five aspartate tags was effectively secreted into the culture medium. The recombinant *E. coli* produced 40.8 U/mL of asparaginase II isoenzyme in the medium during batch fermentation [72].

Furthermore, the combination of in silico and experimental techniques allows the efficient and cost-effective testing of a wide range of signal sequences for secretory synthesis of the enzyme. The OmpA and DsbA signal peptides were selected for secretory expression of the *Erwinia chrysanthemi* L-ASNase in *E. coli*. L-ASNase was translocated across the cytoplasmic membrane using either the DsbA or OmpA signal peptide. DsbA, a co-translational signal peptide, localized asparaginase to the cell membrane more efficiently than OmpA, a post-translational signal peptide [105].

#### 2.1.3. Codon Optimization

The strategy of codon optimization significantly increased protein expression. The volumetric output of recombinant proteins and metabolites can be increased by codon optimization or redesign of novel biosynthetic routes. The result is a cost-effective process and product [68]. Except for Met and Trp, amino acids are encoded using 2–6 synonymous codons, and the choice of codons in coding sequences is not arbitrary. This is known as codon use bias and is found in almost all genomes. Codon use bias has long been thought to control the rate of protein production. Because there are so many tRNAs to use for decoding, highly expressed genes use more codons more often, helping to speed up translation. On the other hand, rare codons accumulate in genes with low expression and slow protein translation [106].

Codon use bias affects the expression of heterologous proteins in *E. coli*. In the first scenario, the researchers replaced the codon sequence of the gene of interest with those found in the host organism and then ordered the modified gene for synthesis. The second technique involves the use of a genetically modified strain of *E. coli* that can effectively translate any codon. Codon optimization has been shown to improve protein production by more than 1000-fold in some experiments. By overcoming the limitations associated with species-specific differences in codon usage and tRNA abundance, codon optimization increases the rate of translation [76].

Codon optimization was used to create the synthetic gene tsA_mod to express recombinant protein more efficiently in *E. coli* cells. The codon adaptation index (CAI) was 0.72, close to the target of >0.8. The final optimized sequence showed a change in GC percentage from 38.5% to 50.18% after codon modification. In addition, the artificial gene tsA_mod was cloned and inserted into the pET-28a(+) vector. For heterogeneous expression of TsA, the constructed plasmids were transformed into the host *E. coli* BL21 (DE3). Bacterial lysates from the recombinant strains were analyzed for L-ASNase activity, and it was found that only the synthetic gene (tsA_mod) promoted heterologous expression and produced a high yield of active enzyme TsA in the host strains [107].

The codons of the synthetic L-ASNase gene from *Zymomonas mobilis* were optimized to match the most commonly used codons in *E. coli*. The optimized Z. mobilis L-ASNase gene was cloned and inserted into *E. coli* using the pET26b and pET28a vectors and linked to a histidine tag. *E. coli* BL21(DE3)/pET26b/ans and *E. coli* BL21(DE3)/pET28a/ans were obtained as two clones. Active L-ASNase was expressed extracellularly in *E. coli* BL21(DE3)/pET26b/ans in much higher amounts than L-ASNase from the original microorganism and was cytotoxic to leukemic cells [79].

#### 2.1.4. Fusion Tags

**Figure 3:**
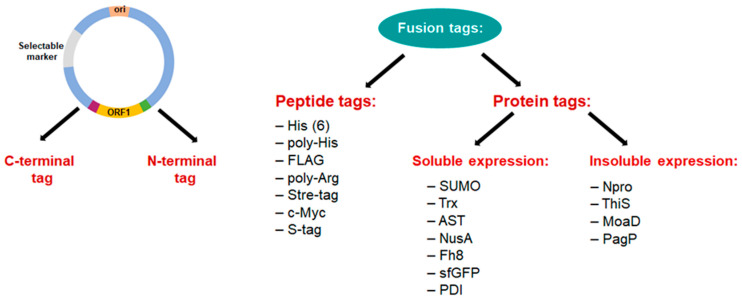
Key tags for the design of an expression vector to enhance heterologous L-ASNase expression *in E. coli*. Fusion tags include: (1) Peptide tags—peptide tags are short amino acid sequences that can be fused to the N- or C-terminus of a recombinant protein to facilitate protein purification and detection; (2) Protein fusion tags—the protein fusion tags are available for soluble and insoluble expression (for intrinsically disordered proteins). Protein tags are larger protein domains that can be fused to the N- or C-terminus of a recombinant protein to improve protein solubility, stability and purification.

Protein expression and/or solubility are enhanced by fusion tags (Figure 3) [108]. These tags, proteins or peptides attached to target proteins aid natural folding, resulting in optimal functional activity or increasing the expression level of an underexpressed protein. Affinity tags are used in conjunction with the associated affinity binding ligand to enable rapid and effective protein purification, resulting in a higher production yield of the produced protein. FLAG, poly-His, poly-Arg, Stre-taq, c-Myc, and S-taq are the most commonly used affinity peptide tags because their small size interferes with the protein of interest. Small ubiquitin-related modifier (SUMO) [109], maltose-binding protein (MBP) [110], N-utilization protein A (NusA), thioredoxin (Trx) [111], glutathione-S-transferase (GST) [112], disulfide isomerase I (PDI), *Fasciola hepatica* antigen (Fh8) and Superfolder green fluorescent protein (sfGFP) are examples of protein tags that improve protein solubility. Examples of insolubility-enhancing tags are N-terminal autoprotease (Npro), β-barrel outer membrane protein (PagP) and two smaller sulfur carrier proteins (ThiS) and (MoaD). Insoluble expression is thought to be more effective than soluble fusion in hiding the toxicity of peptides and protecting them from degradation [113].

Successful overexpression of the 984 bp *Pseudomonas aeruginosa* L-ASNase full-length coding sequence gene (GenBank accession number KU161101.2) was achieved in *E. coli* DE3(BL21) pLysS as a 6-His-tag fusion protein after 18 h induction with lactose at 2 g/L in fermentation medium and at 37 °C. The recombinant enzyme had a specific activity of 19,758.8 U/mg and a purification factor of 10.28 [114].

L-ASNase II of *S. cerevisiae* was expressed in *E. coli* BL21(DE3) to produce a novel modified form without post-translational modifications. Previously, this protein was insoluble and inactive in all but one technique (pET15b; *E. coli* CodonPlus (DE3); amino acids 32–362; induction at 12 °C/0.1 mM IPTG/24 h), where a tiny amount of soluble protein with low specific activity was obtained. The *S. cerevisiae* ASP3 gene encoding L-ASNase II was cloned and inserted into the expression vector pET28a and fused with a 6-fold histidine tag. The resulting recombinant vector (pET28a-ASP3) was used to transform electrocompetent *E. coli* DH5α cells. The plasmid was isolated from desired clones and then used to transform competent *E. coli* BL21 (DE3) cells. L-ASNase was expressed at high levels (225.6 IU/g of cells) as an intracellular and soluble form. The enzyme had relatively low activity toward L-glutamine. The enzyme also showed anticancer activity against K562 and Jurkat cell lines that was equal, if not superior to commercial *E. coli* L-ASNase [115].

The *Saccharomyces cerevisiae* genes (Sc_ASNaseI and Sc_ASNaseII) encoding ASNase were cloned and inserted into *E. coli* in the work of Santos et al. The Sc_ASNaseII protein was always produced in an insoluble and inactive form in all 93 expression conditions evaluated. However, the soluble portion of the protein extract yielded a significant amount of recombinant protein labeled with Sc_ASNaseI (His)6. The specific activity of Sc_ASNaseI was high (110.1 ± 0.3 IU mg^−1^) [116].

L-ASNase mutants of *E. chrysanthemi* with lower L-glutaminase activity and fewer side effects were created by Nguyen et al. A synthetic codon-optimized gene corresponding to the ErA amino acid sequence was cloned and inserted into the His6-SUMO-pET14b vector of *E. coli* (the His6 tag was followed by the yeast protein SUMO (small ubiquitin modifier, Smt3p)). During purification, the N-terminal His6-SUMO tag was cleaved. As a result, the double mutant A31I/E63Q had the best combined properties, retaining 60–90% of wild-type L-ASNase efficiency while reducing L-glutaminase activity by 95% [117]. The authors also discovered that the His-SUMO tag stabilized the ErA mutants in vivo. Through retaining the SUMO tag, the resulting ErA mutants had a longer circulation time. The SUMO tag had little effect on the enzymatic activity of the mutants. Notably, therapy with this tailor-made SUMO-ErA-WT enzyme (50 IU/day ip for 12 days) resulted in a significant reduction in tumor burden in xenografted LOUCY mice. Retention of the SUMO mark increased the stability of the enzyme while having little or no effect on the therapeutic enzymatic capabilities of ErA-WT. As a result, this stability marker was added to both the wild-type and mutant ErA enzymes used in future in vivo drug treatment studies [118].

Fusion of ErA-TM (variant of *E. chrysanthemi* (ErA) with three mutations [117]) with an albumin-binding domain (ABD) tag was shown to significantly increase its persistence in vivo. Furthermore, when the therapeutic efficacy of ABD-ErA-TM was evaluated in vivo in the B-ALL SUP-B15 xenograft model, a long-lasting, sustained anti-leukemic effect comparable to standard pegylated asparaginase therapy was observed, but with fewer acute adverse effects related to glutaminase activity [119].

Recombinant L-ASNase was designed by fusion with the 30Kc19 protein. Due to the presence of a cell-penetrating peptide (CPP) domain in the protein, the 30Kc19 protein has both protein stabilization and cell penetration capabilities. As 30Kc19 can cause steric hindrance, a PLGLAG (LK) linker sequence was inserted between ASNase and 30Kc19. Treatment with the ASNase-LK-30Kc19 fusion protein improved cell penetration, stability and anticancer efficacy. Intracellular transport of both uncleaved and cleaved forms of the protein was demonstrated, indicating that L-ASNase acts both internally and externally, demonstrating significant anticancer activity via effective Asn depletion. In addition, L-ASNase released from ASNase-LK-30Kc19 reached the same half-maximal inhibitory concentration at five times lower concentrations than unreleased ASNase-30Kc19 [120].

Guo et al. created a fusion protein consisting of L-ASNase from *E. coli* and a single-chain protective Fv (scFv) selected from a phage display scFv library from previous work. A linker peptide (Gly(4)Ser)(6) was used to attach the fusion protein antibody fragment to the N-terminus of the enzyme fragment. After refolding and purification, the resulting soluble fusion protein had approximately 82% of the enzymatic activity of native ASNase at the same molar concentration and a K_m_ value similar to the native enzyme K_m_ value for the substrate L-asparagine, and the fusion protein was more resistant to proteolysis by trypsin, alpha-chymotrypsin and rennet than native L-ASNase, which became inactive. The hybrid enzyme maintained 94.0%, 88.8%, and 84.5% of its initial activity. The in vitro half-life of the ScFv-ASNase hybrid in serum was significantly longer (9 h) than that of the native enzyme (2 h) [121].

#### 2.1.5. Co-Expression with Chaperones

Molecular chaperones are ubiquitous and highly conserved proteins that guide the folding of other polypeptides and are responsible for the maintenance of protein folding homeostasis in cells. Molecular chaperones play an important role in protein quality control by helping nascent polypeptides to form their final structure [122]. They can be classified into two groups: molecular chaperones and chaperonins. In heterologous protein expression, chaperones can be used as fusion tags to facilitate protein expression in the soluble fraction and assist in proper protein folding. They can also restore the native structure of the protein [123].

In recent years, numerous studies have been carried out to increase the solubility of recombinant proteins and correct their folding, including the technology of co-expressing proteins with molecular chaperones. The most typical disadvantage of recombinant protein production is the formation of inclusion bodies due to poor protein folding. As a result, the structure—function relationship of a protein can be lost. There are many ways to overcome this problem, but one key strategy is the use of molecular chaperones that act as protein folding modulators. Chaperones are often used to increase the expression of various proteins that are difficult to produce in *E. coli*. In recombinant technology, important *E. coli* molecular chaperones include trigger factor, GroEL, DnaK and ClpB [124,125].

Co-expression of individual chaperone sets has been a common strategy to increase recombinant quality and solubility (Figure 3) [126]. Co-expression of the Pcal_0970 L-ASNase gene from *Geobacillus thermopakistaniensi* with the GroEL molecular chaperone in *E. coli* resulted in the synthesis of the soluble recombinant Pcal_0970 protein. The refolding of Pcal_0970 underwent complete autocleavage after 48 h of incubation at 80 °C, indicating the high heat stability of the enzyme. Pcal_0970 autocleavage was not affected by pH, glycine or metal ions [127].

Although a signal peptide is often required for *E. coli* to efficiently produce foreign proteins in the periplasm, the presence of such a peptide does not ensure that the target proteins will be expressed in the periplasm. Overproduction of auxiliary proteins, such as chaperones, may be a good strategy to improve protein export. In a study by Fin et al., three sets of chaperone plasmids, GroEL-GroES (GroELS), Dnak-Dnaj-GrpE (DnaKJE) and trigger factor (TF), were co-expressed in *E. coli* BL21 (DE3) in pairs with two pET22-b vectors carrying recombinant hirudin-PA (Hir) and various alkaline phosphatase (PhoA) and L-ASNase II signal sequences. DnaKJE was found to be the only factor that significantly increased L-ASNase expression in the periplasm (*p* = 0.04) [128].

Eleven *E. coli* expression strains with different characteristics were evaluated to determine which were best suited for low-temperature (16 °C) expression of recombinant *E. coli* type II L-ASNase (EcAII). ArcticExpress (DE3), a strain co-expressing the *O. antarctica* chaperonins Cpn10 and Cpn60 [82], was used to produce a correctly folded tetrameric recombinant rEcAII according to structural analyses. The remaining strains produced either inactive or misfolded enzymes. In addition, enzymatic tests showed that the proteins produced by ArcticExpress (DE3) had a high specific activity compared to other strains used in this study. The GroEL (DE3) chaperone did not have the same effect on ArcticExpress (DE3) cells, probably due to the reduced activity of GroEL/ES at low temperatures. As a result, only ArcticExpress (DE3) was able to produce rEcAII with a secondary structure content very similar to that of the commercial enzyme. This suggests that the folding of rEcAII can be effectively promoted by the use of auxiliary proteins such as the chaperones Cpn10 and Cpn60 [82].

ClearColi^®^, SHuffle^®^ and other modified strains of *E. coli* are the most commonly used strains for the co-expression of recombinant proteins. Tollabi et al. studied the expression of the Q59L mutant L-ASNase in the presence of chaperones in the *E. coli* SHuffle^TM^ T7 strain. In the presence of chaperones, the amount of soluble recombinant protein increased. The SHuffle^T7^ strain produced more soluble recombinant protein than the BL21 DE3 strain [129].

Except for a few *E. coli* variations such as Rosetta, the *E. coli* expression system typically does not offer post-translational changes. The N- and O-linked glycosylation, hydroxylation, amidation, sulfation and palmitoylation processes are not supported by the *E. coli* expression system. In comparison to *E. coli*, the yeast expression system is more sophisticated because it produces disulfide bonds more effectively [130]. Many recombinant proteins require the formation of specific disulfide bonds to achieve a physiologically active three-dimensional structure. Incorrect disulfide bond formation can lead to protein misfolding and aggregation in inclusion bodies (IBs). Cysteine oxidation occurs in the periplasm of *E. coli*, where disulfide bonds are formed during disulfide exchange events. In contrast, disulfide bond formation in the cytoplasm is rare, probably because cysteine residues are found in the catalytic centers of many enzymes. Disulfide bond formation at these sites can lead to protein inactivation, misfolding and aggregation. However, cytoplasmic expression is still possible thanks to modified *E. coli* strains with an oxidative cytoplasmic environment that favors the formation of disulfide bonds. Of particular interest are the Origami (Novagen) and Shuffle (NEB) strains. The Origami^TM^ strain has the trxB gor genotype and is available in the BL21(DE3) lacY background (Tuner^TM^, Novagen). With the inclusion of the pRARE plasmid for the additional benefit of codon bias correction, the Rosetta-gami^TM^ B strain (Novagen) was created. In addition to the trxB and gor mutations, the SHuffle^®^ T7 Express strain (BL21(DE3) background, NEB) constitutively expresses a chromosomal copy of DsbC (disulfide bond isomerase). DsbC is a chaperone that can stimulate the folding of proteins that do not require disulfide bonds and helps to repair misoxidized proteins to their correct shape. As a result of DsbC activity, less of the target protein aggregates in IB [73].

Genetic screening for expressing soluble proteins in *E. coli* was performed by Vernet et al. Low-expressing constructs of 11 targets and the *E. coli* L-ASNase II construct were cloned and inserted into expression vectors for secretion into the periplasmic space or into the culture medium, as several targets were insufficiently expressed. Export of the recombinant protein to the periplasm or extracellular space using the secretion vectors pNIC-BASY (OsmY fusion), pNIC28-DsbA1 (DsbA fusion) and pNIC28-pelB1 (pelB secretion using the pNIC28-DsbA1 vector) helps to avoid the problem of nonexistent or incorrect disulfide bond formation. Using the pNIC-BASY vector, 23 out of 49 constructs (or 47%) produced soluble protein, whereas similar figures for pNIC28-DsbA1 and pNIC28-pelB1 were 22/35 (or 63%) and 4/26 (15%), respectively [131]. 

The N-terminal domain of *Pyrococcus furiosus* L-ASNase (NPfA) is a novel noncanonical molecular chaperone with a molecular mass of 22 kDa. This domain was generated by truncating the C-terminus of *Pyrococcus furiosus* L-ASNase. This enzyme and its mutant have been shown to have anticancer activity. NPfA differs significantly from the known small molecular chaperones, which have strong sequence similarities to α-crystallin. However, its function is similar to that of small heat shock proteins (sHSPs), and it acts not only as an internal chaperone but also as a molecular chaperone, preventing thermal aggregation of various proteins in vitro. In the absence of protein stability and an increase in membrane permeability, *E. coli* growth is inhibited above 44–46 °C. Such limitations notwithstanding, heterologous expression of chaperone molecules has the potential to augment the thermotolerance of *E. coli* [132]. In a study by Jena et al., NPfA, a noncanonical, non-natural chaperone, was shown to confer a selective growth advantage in *E. coli* cells under extreme temperature conditions. Recombinant expression of NPfA allowed *E. coli* to grow at 52 °C and survive heat shock up to 62 °C [133].

#### 2.1.6. Optimization of L-Asparaginase Production in *Escherichia coli*

Recent molecular research has focused on a variety of issues, including expression vector design, gene copy number optimization, co-expression of secretory proteins such as chaperones, glycosylation and secretory pathway engineering. However, it is often difficult to distinguish the physiological effects of different culture tactics from the molecular consequences of the gene construct (e.g., cellular stress caused by overexpression or faulty post-translational processing). As a result, overall system optimization is difficult, although it is critical to describing and understanding the behavior of new molecular constructs [134].

L-ASNase production is greatly influenced by the composition of the fermentation medium and culture parameters such as temperature, pH, inoculum size, agitation rate and incubation time [135]. Thermal stability is also important as it improves the shelf life of the protein and provides long-term storage stability [136]. The majority of microorganisms reported optimal L-ASNase production temperatures between 25 and 37 °C. In fact, the temperature of the culture medium directly affects the growth of the microorganisms, which in turn affects the production and activity of the enzymes [137]. 

The optimum temperature of the recombinant enzyme varies depending on the bacterial source and the expression host [138]. The optimal pH and temperature for L-ASNases from *E. coli* MTCC739 in *E. coli* BL21(DE3) are 37 °C. For example, the optimal temperature and pH for rASPG from the thermotolerant strain *E. coli* KH027 in *E. coli* DH5 were 43 °C and pH 6. For rASPG from *E. coli* W3110 in *E. coli* BL21(DE3), the conditions were 37 °C and pH 7.5 [138]. According to research by Borah et al. (2012), L-ASNase can be produced from *E. coli*, with 55 °C being the optimal temperature for enzyme activity [139]. Goswamia R. et al. investigated the best conditions for higher recombinant L-ASNase II activity of *Erwinia carotovora* subsp. *atroseptica* SCRI 1043 in *E. coli*. The pH and temperature of the process parameters were optimized using response surface methodology (RSM) to investigate the optimal settings for recombinant enzyme performance. Thermodynamic parameters for recombinant L-ASNase II were determined and the enzyme is more stable at pH 8.5 at 35 °C than at pH 6.5, 7.5 and 9.5 [140].

The primary purpose of separating growth and production is to maximize host resources. The basic strategy is to decouple cell growth from recombinant protein production. Host cells are initially cultured at normal growth rates, without competition from recombinant protein production. Once the culture has reached a stable stage, growth is stopped, and recombinant protein production is stimulated so that most of the resources are used to synthesize the product. The autoinduction system is widely used in industrial production. Glucose, lactose or glycerol are commonly added to traditional autoinduction media. To broaden the range of applications and reduce expression leakage, many forms of autoinduction systems have been developed based on concepts such as quorum sensing, phosphate induction or molecular chaperones that can unblock catabolite repression in *E. coli* [88].

The majority of commercial enzymes are produced from expensive simple sugars in submerged liquid fermentations (SmF). Solid-state fermentation (SsF) can also effectively produce enzymes from solid organic residues, but difficulties in developing and scaling up SsF technologies, particularly bioreactors, have limited the use of such systems. SmF is widely used for the large-scale production of L-ASNase. In the SmF process, microorganisms grow in a liquid broth medium supplemented with the nutrients necessary for optimal microbial cultivation. The selected microorganisms were carefully cultivated in a closed reactor containing fermentation medium and a high level of oxygen [141].

To optimize the extracellular production of recombinant L-ASNase in *E. coli* in the shaker and bioreactor, an exponential feeding strategy was used to maintain a specific growth rate of 0.3 h^−1^. To investigate the effect of induction time on expression, cultures were induced with 1 mM IPTG at various optical densities and maintained in culture medium throughout the incubation. The feed was gradually increased until the period of biomass increase after induction. However, as the specific growth rate dropped abruptly a few hours after induction, the feed was kept constant and then gradually reduced using an indirect feedback technique involving constant, level management of dissolved oxygen (DO), i.e., DO-stat (DO-stat). As a result, the amount of extracellular recombinant protein was 54% of the total extracellular protein 12 h after induction and 42% after 24 h. Although the rate of specific product production decreased with the increase in OD of induction, induction at an OD600 of 90 resulted in a maximum volumetric activity of 8.7 × 10^5^ U/L, equivalent to 5.24 g/L of recombinant L-ASNase [93].

The synthesis of recombinant L-ASNase II from *E. coli* BL21(DE3) in a shaker and 3 L bioreactors was optimized by Barros et al. The activity of L-ASNase was assessed using three different culture media: defined, semi-defined and complicated. Lactose was used as an inductor for the bioreactor tests. Batch and fed-batch cultures were modified to achieve high cell densities prior to induction. L-ASNase was synthesized in a shaker and then scaled up to a bioreactor, resulting in a 27-fold increase in cell concentration and enzyme yield. When the cell concentration was scaled up to the bioreactor, it increased 27-fold, reaching approximately 70 g/L dry weight and 43,954.79 U/L volumetric activity [142].

A culture approach based on enzymatic delivery of glucose was introduced to overcome the drawbacks of growing *E. coli* L-ASNase in shaking flasks (low cell yield and insoluble or misfolded target protein). This technique, called EnBase technology, mimics the fed-batch approach used in bioreactor culture and produces high yields of biomass and recombinant protein during shaking culture. EnPresso B medium can provide many times higher yields of recombinant proteins and biocatalysts per volume of culture compared to LB and other traditional growth media [143].

Traditionally, fermentation-based biological product optimization has been carried out using the one-factor-at-a-time (OFAT) technique, where numerous physiological and nutritional parameters and their varying ranges have been found to have a significant impact on enzyme synthesis [144]. However, numerous studies have shown that statistical modeling approaches are more effective than other methods in maximizing enzyme production in bioprocesses by minimizing errors, identifying true effects, taking into account interactions between different parameters, etc. Statistical optimization approaches, such as Plackett-Burman (PB) and response surface methodology (RSM), have been used in several studies to determine the actual effects and interactions between parameters on L-ASNase production from various microorganisms [144,145]. 

OFAT and RSM were used to optimize and produce L-ASNase from various processes and media characteristics of *P. resinovorans* IGS-131 by changing one factor at a time while keeping the rest constant. Temperature (25–45 °C), pH (4–9), inoculum percentage (1–10% of 1 × 10^8^ CFU/mL), agitation speed (100–350 rpm), incubation time (6–48 h) and production medium were studied as physiological parameters. L-ASNase production was further optimized using response surface methodology (RSM) with a central composite design. The most significant variables (Na_2_HPO_4_, KH_2_PO_4_, NaCl, L-asparagine and % of inoculum) were set at five different levels in the RSM model. A set of 26 experiments with five replicates was constructed in a CCD matrix. The use of conventional and statistical optimization techniques to optimize L-ASNase production resulted in an enzyme yield of 37.63 IU/mL, which was 3.45 times greater than the initial enzyme activity (i.e., 10.91 IU/mL). A study on the effect of agitation during enzyme production in a bioreactor showed a maximum L-ASNase yield of 38.88 IU/mL after 24 h of fermentation at 400 rpm [144].

Medium optimization using RSM for the production of L-ASNase II in *E. coli* was successfully performed by Ghoshoon et al. The maximum yield of L-ASNase in the flask was 17,386 U/L with the optimized medium components (7.75 g/L tryptone, 5.25 g/L peptone, 9 g/L yeast powder, 0.6 g/L calcium chloride), which was only 1.7% different from the predicted value of the model, suggesting an alternative choice for better optimization of the fermentation process [146].

RSM was also applied to identify the most important variables and then optimize the environment for the synthesis of L-ASNase from an actinomycete strain (CA01). In a basic medium containing (g/L): K_2_HPO_4_ (0.5), MgSO_4_ (0.5) and 7H_2_O (0.1), the ideal conditions for the production of the enzyme resulted in an optimal enzymatic activity of 8.03 IU/mL at 27.83 °C. The enzyme reached maximum production on the sixth day of the experiment. The model developed for the production of active L-ASNase by strain CA01 is significant under the following linear conditions: temperature, substrate concentration and glucose concentration [147].

Overexpression of recombinant proteins induces a cellular stress response (CSR) that suppresses the expression of several genes. Transcriptome analysis of postinduction *E. coli* cultures expressing recombinant L-ASNase revealed the downregulation of several critical genes, many of which were controlled by the global regulator lrp, known to have a significant effect on both amino acid metabolism and protein translation. To counteract the negative effects of CSR, plasmid-based co-expression increased lrp activity. After 32 h of batch culture, a test culture with an additional plasmid expressing lrp under an arabinose promoter yielded 50% more recombinant L-ASNase than a control culture with only one plasmid expressing the recombinant protein [148].

The combination of gene deletion with the downregulated expression of important genes may mitigate the negative consequences of CSR. Genes with no known downstream regulators were identified, and knockouts were made and evaluated for L-ASNase and GFP expression. To further understand the function of these genes, knockouts were made in two different host systems: *E. coli* BW25113 and W3110. Many of these knockouts showed much higher levels of expression that persisted for longer periods of time. Of the 45 double knockouts developed, BW25113ΔelaAΔyhbC and BW25113ΔcysJΔyhbC had the best yield increase over single gene mutant strains [149].

### 2.2. Bacillus subtilis Expression System

*B. subtilis* is a nonpathogenic and GRAS (generally regarded as safe) bacterium. The main advantage of *B. subtilis* is that it does not produce LPS, which can cause degenerative diseases in humans and animals. Because of its genetic characteristics, *B. subtilis* can also be easily modified using a variety of bacteriophages and plasmids. It can secrete functional extracellular proteins directly into the culture medium, facilitating subsequent purification steps. It has no major bias in codon usage, which is considered an advantage [150]. Compared to the *E. coli* expression system, *B. subtilis* has negligible protein secretion potential, and little information is available on the production of L-ASNases by *B. subtilis* [40].

There are many strains of *B. subtilis*, including auxotrophic strains 23, 122, 160, 166 and 168, which were initially developed from *B. subtilis*. The 168 strain has been widely employed in academic and industrial research. To obtain mutant strains that meet production needs, several strains can be altered through systematic metabolic engineering. For the production of heterologous proteins, *B. subtilis* WB600 and WB800 strains were created by the removal of six and eight protease genes, respectively, from *B. subtilis* 168 [151].

Expression systems for the production of recombinant proteins in *B. subtilis* can be created using either independent plasmid expression or expression from a chromosome-integrated expression cassette. The *B. subtilis* expression system has some disadvantages compared to the *E. coli* expression system, including unstable plasmids, lack of plasmid diversity and poor plasmid transformation rates. Plasmid stability and copy number can usually be altered by introducing a replication gene [152]. There are few standardized promoters documented in the *B. subtilis* registry compared to *E. coli*, and most are constitutive elements [153]. Insertion of the ori region has been shown to promote plasmid replication in *B. subtilis* [154]. Expression plasmids with improved structural stability have also been generated from θ-replication plasmids [155]. When using single plasmids to express recombinant proteins, multicopy plasmids are required [156].

Several expression systems have been developed in different *Bacillus* species to overproduce heterologous proteins [157]. Constitutive promoters, double promoters, functional synthetics capable of directing transcription, effective signaling peptides, inducible and self-inducing expression systems and post-transcription regulation can improve the expression level of recombinant proteins in *B. subtilis* [151,152].

#### 2.2.1. Promoter Engineering

Using modern genetic techniques, such as “Omics”, numerous novel promoters have been discovered in *B. subtilis* throughout the years. There are several native promoters that regulate endogenous genes in the large collection of inducer-free promoters, some of which have higher transcriptional activity than others. These include the rpsD promoter, the P43 promoter, lepA and the vegI promoter. The most widely used promoter in *B. subtilis*, the P43 promoter, has been fully characterized, verified and used in recombinant expression systems to overexpress heterologous proteins with various functions [153].

The use of dual promoters is a promising strategy for increasing the productivity of recombinant proteins in *B. subtilis*, which has attracted considerable attention due to its high efficiency and consistency [158]. A dual promoter system was used in a study by Niu et al. to enhance the production of type I L-ASNase from *Bacillus licheniformis* Z-1 (BlAase) in *B. subtilis* 168. The original P43 promoter was first replaced by fifteen single strong promoters. The maximum BlAase activity (436.28 U/mL) was obtained using the PyvyD promoter. To increase BlAase production, dual promoter systems were constructed using four promoters (PyvyD, P43, PaprE and PspoVG) that had comparably high levels of BlAase expression. The activity of the dual promoter, PaprE-PyvyD, reached 502.11 U/mL. By altering key regions of the PaprE-PyvyD promoter, BlAase activity was similarly increased (568.59 U/mL). BlAase activity increased to 790.1 U/mL, which is 2.27 times higher than that with the original PyvyD promoter ribosome-binding site (RBS) sequence from the P43 strain. The expression level of BlAase in a 10 L fermenter after 36 h of incubation was 2163.09 U/mL, which is 6.2 times higher than that of the original strain with the P43 promoter. In addition, an 89.50% reduction in acrylamide was achieved when BlAase was used to reduce acrylamide in potato chips. These results suggest that using transcription and translation processes in tandem is a successful method for increasing the productivity of recombinant proteins and that BlAase could be a top contender for acrylamide management in the food sector [159].

A number of techniques were used to increase L-ASNase production, including signal peptide optimization, N-terminal truncation and selection of fermentation conditions. The *B. subtilis* 168 L-ASNase II gene was successfully produced in *B. subtilis* WB600 after coupling to the expression vector pP43NMK with a strong P43 promoter. The wapA signal peptide was selected from the L-ASNase signal peptide, ywbN, yvgO, amyE, oppA, vpr, lipA and wapA, which were used for L-ASNase secretion in *B. subtilis* using the Hpa II promoter. The highest extracellular L-ASNase activity (28.91 U/mL) was achieved with the wapA signal peptide. L-ASNase activity was increased by 38.1% by further replacement of the Hpa II promoter with the strong P43 promoter. Two rounds of PCR were used to identify P43 promoter variants with significantly improved potency, including B2 (-28: A → G, -13: A → G), which increased L-ASNase activity to 51.13 U/mL. L-ASNase activity increased by 100% over intact enzyme to 102.41 U/mL following deletion of the N-terminal 25 residues. The maximum activity of purified L-ASNase was observed at 65 °C, where the half-life was 61 min. The corresponding K_m_ and k_cat_ L-ASNase values were 5.29 mM and 54.4 s^−1^, respectively. Thus, the bacterial host produced a very high yield of L-ASNase for industrial use [160].

#### 2.2.2. Plasmid Engineering

Plasmids pUB110, pLS1, pE194, pAM1, pSB6, pTB19, and pC194 are the main sources of *B. subtilis* expression plasmids [152].

Overexpression of L-ASNase (ansZ) was obtained from a nonpathogenic strain of *B. subtilis* B11-06 in a study by Zhia et al. Using the pMA5 shuttle vector, the ansZ gene encoding L-ASNase II was expressed in *B. subtilis* 168. The recombinant pMA5-ansZ plasmid was then transformed into the *B. subtilis* 168 expression host to create a recombinant *B. subtilis* 168/pMA5-ansZ strain expressing ansZ under the HpaII promoter. Positive clones had intracellular and extracellular enzyme activities of 4.28 and 5.70 U/mL, respectively. The total enzyme activity was much higher than that of the native *B. subtilis* B11-06 strain. The recombinant enzyme was thermally stable and had a low affinity for L-glutamine. The activity of the recombinant enzyme was 9.98 U/mL, which was significantly higher than that of *B. subtilis* B11-06. The K_m_ for L-asparagine was 0.43 mM, and the Vmax was 77.51 μM/min [161].

A synthetic system for L-ASNase of *B. subtilis* was constructed by Li et al. The plasmid pMA5-S17G/A90S/R156S/K272A was designed to include the mutant *Pyrococcus yayanosii* CH1 L-ASNase gene. For gene cloning, the pMA5 plasmid was utilized as a template, with the PHpaII promoter followed by the RBS sequence from pMA5. P43, PgroEs, PsigX, PtrnQ and PyxiE promoters were used to amplify genes from *B. subtilis* 168 genomic DNA, followed by the RBS sequence from pMA5. The P43 promoter genes contained both their natural RBS sequence and synthetic RBS207 and RBS206 sequences. The Pshuttle-09, P131 and P242 promoter genes were cloned and inserted into the pUCk plasmid using the RBS sequence from pMA5. Through promoter screening and RBS design, the transcription level and translation rate of the *Pyrococcus yayanosii* CH1 ansB gene in *B. subtilis* were enhanced. The resulting L-ASNase activity after fermentation optimization was 5278 U/mL [26].

A novel engineered *B. subtilis* strain was tested for the synthesis of *Aliivibrio fischeri* L-ASNase II. Genetic modification was carried out using the constructed pBS0E plasmid replicative in the new *B. subtilis* KO7 (BGSCID 1A1133) strain and carrying the xylose-inducible PxylA promoter and its repressor sequence (XylR). The results showed that a stirred tank bioreactor could produce L-ASNase with activity up to 1.539 U/mL. The enzymatic extract had a pH of 7.5, high L-asparagine affinity (K_m_ = 1.2275 mmol L^−1^) and low L-glutaminase activity (0.568–0.738 U mL^−1^). In addition, low kinetic thermal inactivation constants for 25 and 37 °C (0.128 and 0.148 h^−1^, respectively) indicate higher stability [162].

The gene for glutaminase-free L-ASNase II of *Pectobacterium carotovorum* MTCC 1428 was cloned in pHT43 and transformed into *B. subtilis* WB800N, and the expression levels of the recombinant enzyme were optimized. The pHT43 vector used in the study consisted of a Bacillus signal peptide (amy Q) linked to a Shine-Dalgarno sequence, allowing amplification of the asnB II gene without the native signal peptide. The resulting pHT43-ans B2 vector was transformed into *E. coli* DH5α and grown in medium. The specific activity of the recombinant protein was 101 IU/mg. Enzyme localization studies showed that more than 90% of the recombinant enzyme was released extracellularly [163].

#### 2.2.3. Enhancement of Protein Secretion Level

The expression of recombinant proteins in *B. subtilis* is relatively straightforward. Specific signal peptides are present at the N-terminus of each recombinant protein and must be cleaved by a peptidase to release the mature protein for export to the medium, retention in the cell wall or retention at the cell membrane interface. Signal peptides are required to properly export the protein. Many studies have been conducted to explore the properties of signal peptides associated with efficient protein secretion, and many papers have demonstrated that the use of an appropriate signal peptide is critical for the highly efficient production of extracellular proteins [151].

According to Niu et al., L-ASNase from *Bacillus licheniformis* Z-1 (BlAase) may act as a signaling peptide that drives the secretion of other proteins via a nonclassical secretion pathway. The BlAase was successfully cloned and produced in *B. subtilis* RIK 1285. The effective secretion of BlAase suggests that it could act as a signal peptide for the export of other recombinant proteins from *B. subtilis*. Several heterologous proteins, including *B. licheniformis* Z-1 type II L-ASNase (BlZ, extracellular protein), *B. megaterium* H-1 type I L-ASNase (BmA, extracellular protein), groES, grpE (cytoplasmic proteins) and prsA (membrane protein) from *B. subtilis* 168, were selected and fused to the C-terminus of B1Aase. Signal peptide prediction using SignalP 5.0 revealed that BlZ, BmA and prsA all have a signal peptide at the N-terminus, which was first deleted in the study to eliminate the influence of native signal peptides on fusion protein release. Expression and secretion of the fusion proteins showed that BlAase-prsA and BlAase-grpE were successfully expressed and secreted into the medium, whereas BlAase-groES, BlAase-BlZ and BlAase-BmZ were either expressed as inclusion bodies or not expressed at all. This was the first time that extracellular production of L-ASNase was achieved in *B. subtilis* using BlAase as a signal peptide via a nonclassical protein secretion mechanism [164].

The open reading frame encoding L-ASNase from *Bacillus cereus* BDRD-ST26 (BcA) was expressed in *B. subtilis* WB600 to achieve high production efficiency. The P43-BcA vector was constructed by inserting the amplified BcA gene into the KpnI and PstI restriction sites of pP43NMK and driven by the P43 promoter. Three signal peptides (amyE, lipA and wapA) were cloned from the *B. subtilis* genome to increase the efficiency of BcA secretion in *B. subtilis*. P43-amyE-BcA, P43-lipA-BcA and P43-wapA-BcA recombinant vectors were generated by PCR followed by ligation between identical restriction sites in pP43NMK. The highest yield, 374.9 U/mL, was obtained using the amyE signal peptide. Purified BcA had a specific activity of 550.8 U/mg and had very low L-glutaminase activity. The half-life at 50 °C was 17.35 min, k_m_ and k_cat_ were 9.38 mM and 63.6 s^−1^, respectively, and the maximal activity of BcA was found at pH 9.0 and 50 °C. Potato strips treated with BcA contained 72% less acrylamide (2.35 mg/kg) than untreated potato strips [165].

#### 2.2.4. Optimization of L-Asparaginase Production in *Bacillus subtilis*

The production of recombinant proteins in *B. subtilis* is achieved via SmF or SSF. The production of recombinant proteins in *B. subtilis* is often improved via fermentation optimization, which includes optimization of fermentation media, fermentation process conditions and a feeding strategy. Before achieving optimal fermentation parameters, fermentation media and conditions are usually improved. Some models (such as RSM and artificial neural networks) have also been used in this process [152].

L-ASNase was extracted from a soil isolate of *Bacillus* sp. (DKMBT10) using a SmF technique. A full loop of a log-phase bacterial culture was added to the culture medium and incubated for 24 h on a rotary shaker at 37 °C and 200 rpm. After 18 h of deep fermentation using glucose and maltose as carbon sources, the strain reached maximum development. The purified enzyme showed a higher specific activity with glucose as the carbon source (1.12 U/mg) compared to maltose (1.05 U/mg) after fermentation and a higher maximum synthesis of the enzyme after 24 h of fermentation, according to a study of the structure of enzyme production during fermentation [166].

In a synthetic system for producing L-ASNase in *B. subtilis* based on the P43 promoter and the synthetic RBS sequence, the yield in a shake flask was 371.87 U/mL, which is 2.09 times higher than that of the original strain. To further increase productivity in the 5 L fermenter, a multistage stirring speed method was used. After 42 h of fermentation, the maximum production of L-ASNase was observed to be 5321 U/mL [26].

OFAT was used to optimize the process parameters for the production of L-ASNase II from *Pectobacterium carotovorum* MTCC 1428 in *B. subtilis* WB800N. RPM (120), temperature (37 °C), IPTG (1 mM) and induction time (0.8 OD600 nm) all played an important role in achieving maximum enzyme synthesis of 55 IU/mL. In addition, successive induction with IPTG increased the enzyme synthesis to 105 IU/mL with a specific activity of 101 IU/mg protein [163].

### 2.3. Pichia pastoris Expression System

*P. pastoris* is an effective platform for the production of high-titer recombinant proteins. *P. pastoris* is a methylotrophic yeast typically grown using dynamic culture methods. It has certain unique physiological characteristics, such as the ability to grow rapidly at high cell densities in basal media and to produce proteins in high yields under bioreactor conditions. 

**Figure 4:**
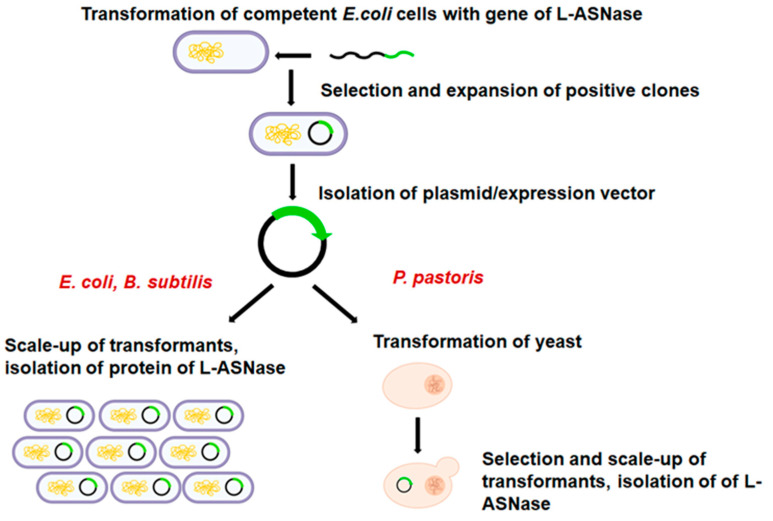
The following are the critical steps in the production of heterologous L-ASNase in *P. pastoris*: (1) the L-ASNase gene (cDNA) is extracted from sample and inserted into the host vector; (2) *E. coli* is commonly used to amplify the constructs; (3) yeast cells are transformed with a vector encoding the cDNA for the L-ASNase gene and colonies are grown on selective medium. This step is different in bacteria.

The advantages of protein production using the *P. pastoris* system include improved folding efficiency, high cell density fermentation, a powerful expression system, genetic stability and a mature secretion system for secreting proteins into the external environment [167]. In contrast to bacteria, linearized foreign DNA can be efficiently incorporated into a chromosome by a process called cross-recombination, to create stable yeast cell lines (Figure 4) [168]. Furthermore, *P. pastoris* is a suitable bacterium for the secretory synthesis of recombinant proteins directly into the culture medium supernatant. Due to the minimal synthesis of endogenous secretory proteins in the *P. pastoris* expression system, the purification of recombinant proteins is straightforward [169]. *P. pastoris*, like Saccharomyces, benefits from molecular and genetic engineering, but the expression levels of heterologous proteins are 10 to 100 times higher, making *P. pastoris* more popular [170]. However, *P. pastoris*, unlike *S. cerevisiae*, is not a fermentative yeast. As a result, almost all glucose is converted to biomass. Because glucose is not converted to ethanol or other organic acids, *P. pastoris* can grow to high cell densities under aerobic conditions [171].

A variety of *P. pastoris* expression vectors and host strains are available [172]. Commonly used *P. pastoris* vectors for the expression of heterologous proteins are pGAPZ, pHIL-S1, pPIC9K, pJL-SX, pBLHIS-SX, pPICZ, pHIL-D2, pJL-IX and pBLHIS-IX. Expression vectors in *P. pastoris*, such as shuttle vectors in *E. coli*, can be transferred between two different host species. In addition, the vectors contain a drug resistance gene, such as Kan, Shble, Bsd, Amp or FLD1, which is resistant to geneticin, zeocin, blasticidin, ampicillin and formaldehyde [169]. The *P. pastoris* strain GS115 is a commonly used strain for protein expression. It has two genes encoding alcohol oxidase enzymes, AOX1 and AOX2. This yeast first attracted attention for its inherent ability to use methanol as its sole carbon supply, which can be achieved through specific metabolic pathways involving numerous specialized enzymes. Over 5000 proteins have been successfully cloned and produced using the *P. pastoris* method. However, *P. pastoris* is unable to synthesize or secrete all the proteins in such high concentrations. Protein production is significantly reduced under normal conditions, especially when complex hetero-oligomeric proteins are produced [167]. The KM71 strain is a derivative of the GS115 strain and has the AOX1 gene deleted, making it a MutS strain [173]. The X33 strain is a prototrophic strain, which means that it can grow on minimal media without the need for any additional nutrients. The X33 strain is Mut+, which means that it is able to use methanol as a carbon source and has a functional AOX1 gene [174]. Other strains, such as SMD1163 (his4 pep4 prb1), SMD1165 (his4 pep4), SMD1168 (his4 pep4), BG21 and PichiaPink^TM^, have no protease, which prevents the degradation of a secreted protein [175].

For intracellular and secretory expression, Life Technologies offers a range of standard *P. pastoris* expression vectors with constitutive (P_GAP_) and inducible promoters (P_AOX1_, P_FLD_). By using ade2 knockout strains and truncated ADE2 promoters of different intensities in front of the ADE2 marker gene, the PichiaPink^TM^ expression kit for intracellular or secretory expression facilitates the selection of multicopy integration clones. BioGrammatics also offers GlycoSwitch^®^ vectors designed for humanized glycosylation of target proteins and holds licenses for the sale of standard *P. pastoris* expression vectors and strains [174]. Some commonly employed Pichia GlycoSwitch^®^ strains are SuperMan5, SuperMan5HIS−, SuperMan5pep4–, and SuperMan5(aox1–, Muts) [169].

In *P. pastoris*, selectable markers are used to identify cells that have taken up the recombinant DNA and to maintain the recombinant DNA in the cells during growth and division. The commonly used selectable markers in *P. pastoris* include: (1) HIS4: the HIS4 gene is a histidine biosynthesis gene that is used as a selectable marker in the transformation of *P. pastoris* cells; (2) URA3 and URA5: the URA3 and URA5 genes are also often used as selection markers in *P. pastoris* (3) ADE1, ARG4 and MET2: these genes are also used as selectable markers in *P. pastoris*. The choice of selectable marker depends on the specific application and the availability of the appropriate auxotrophic strains [176,177].

**Figure 5:**
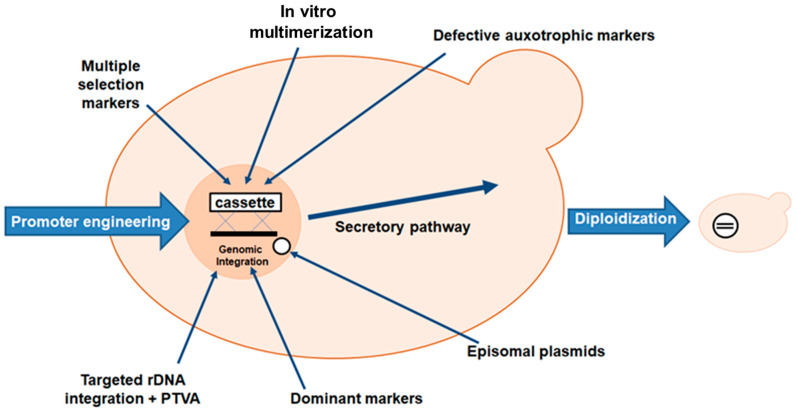
Strategies used in *P. pastoris* to enhance the transcription of the gene of interest (L-ASNase). The titer of heterologous transcripts can be increased by: (1) promoter engineering; (2) introduction of multiple copies of the heterologous gene to obtain multi-copy clones; (3) in vitro construction of multimers of the expression cassette to obtain multi-copy clones; (4) use of dominant markers in multiple clones to increase drug resistance. The use of dominant markers is the basis of the post-transformational vector amplification (PTVA) method; (5) use of defective auxotrophic markers; (6) diploidization of selected clones; (7) combinatorial engineering of secretion helpers involved in protein folding and vesicle trafficking.

Many researchers have used molecular techniques to increase protein production due to the low specific productivity of *P. pastoris*. The most common method of achieving this goal is to deliver increased titers of a particular transcript to the cell’s translation machinery. This is usually achieved by using a suitable promoter to stimulate production of the heterologous gene or simply by increasing the copy number of the target gene (Figure 5) [178]. Pan et al. also described some other approaches, such as host strain engineering by improving homologous recombination efficiency, using episomal plasmids for heterologous gene expression and the CRISPR/Cas9 system for genome editing, selective marker and marker recycling and metabolic engineering [167]. Codon optimization, introduction of artificial glycosylation sites, signal peptide engineering, and cell surface display have also been suggested [173].

One of strategies to enhance the transcription of the gene of interest (Figure 5), in which gene expression cassettes can be incorporated multiple times into the genome, was used for the production of L-ASNase. L-ASNase (GenBank accession number KF290772.1) from *R. miehei* was provided as a component of the widely used pCC1 cloning vector and generated in *P. pastoris*. Six clones carrying different numbers of gene copies (1–5 and 5+) were used to study the effect of gene copy number on the increase in protein production to increase the amount of L-ASNase. According to the findings, the clone with three integrated copies of the expression cassette had the maximum production level [179]. Strategies used to enhance L-ASNase production in *P. pastoris* include promoter engineering, fusion tags, signaling sequences, metabolic engineering and optimization of culture conditions.

#### 2.3.1. Promoter and Vector Engineering

Promoters commonly used in *P. pastoris* include AOX1, GAP, FLD1, ICL1, YPT1 and NPS [172]. In *P. pastoris*, the promoter of the alcohol oxidase I (AOX1) gene is extensively used for recombinant protein expression. The alcohol oxidase enzyme encoded by AOX1 catalyzes the conversion of methanol to formaldehyde in the methanol utilization pathway. *P. pastoris* AOX1 is a potent and tightly controlled methanol-inducible promoter [180]. Because alcohol oxidase has a low affinity for oxygen, large amounts of alcohol oxidase are produced to compensate. The high level of expression of the AOX1 promoter makes *P. pastoris* suitable for the production of recombinant protein [181]. AOX1 has been incorporated into a number of commercially available *P. pastoris* expression vectors, which require the following components: 5′-AOX1 (the alcohol oxidase promoter upstream of the gene of interest); SIG (a secretion signal sequence); MCS (a multiple cloning site); TT (a transcription termination site); HIS4 (a marker for selection by hydroxyhistidinase); Amp (for selection with ampicillin); and ColE1 (a replication element for plasmid propagation in *E. coli*) [172].

In some cases, natural promoters have shown a poor ability to control expression levels and regulatory properties, and several promoter development projects have been attempted for *P. pastoris*, particularly for P_AOX1_. In addition to P_AOX1_, numerous other methanol-induced promoters have been identified in *P. pastoris*. However, in the food and pharmaceutical industries, the use of methanol for the production of high-quality proteins is impractical. Several methanol-free expression systems have been developed for specific applications [167]. Thus, other promoters, such as GAP, FLD1, PEX8 and YPT7, can be used. The GAP promoter, for example, is derived from the *P. pastoris* glyceraldehyde-3-phosphate dehydrogenase (GAP) gene [182]. The advantage of using this promoter is that methanol is not required for induction, and cultures do not need to be transferred from one carbon source to another, making strain development and protein expression more practical and straightforward [172]. The GAP gene can be constitutively expressed in cells maintained on glucose, glycerol or methanol. Compared to glucose medium, the level of GAP promoter activity was two-thirds in glycerol medium and one-third in methanol medium. As a result, the GAP promoter is a viable option for recombinant protein production in *P. pastoris* [181]. However, the GAP promoter is constitutively expressed; therefore, it is not a good choice for producing toxic proteins in yeast [172].

The ADH3 promoter of the alcohol dehydrogenase (ADH) gene is a powerful alternative to the AOX1 and GAP promoters. The enzyme alcohol dehydrogenase (ADH) is responsible for ethanol metabolism. *P. pastoris* can use ethanol as a carbon source as well as glucose, methanol, glycerol, sorbitol, mannitol and others. Two *P. pastoris* alcohol dehydrogenase (ADH) genes, ADH and ADH3, have been identified. In *P. pastoris*, ADH3 is responsible for ethanol utilization, whereas the ADH gene has no function in ethanol production or utilization [181].

Although the choice of protein expression promoters in *P. pastoris* is limited to PAOX1 or PGAP, researchers have made efforts to identify new synthetic promoters that are likely to replace conventional promoters. Engineered promoter variants outperform natural promoters and enable “green and clean” production on a nontoxic carbon source in the first-choice utilization pathway of the yeast carbon source [167]. In recent years, promising new constitutive (PGCW14), regulatory (PGTH1, PCAT1) and bidirectional (histone promoters and synthetic hybrid versions) promoters have been identified. As natural promoters have thus far shown limited tunability of expression levels and regulatory properties, several promoter engineering initiatives have been undertaken for *P. pastoris*. PAOX1, PDAS2, PGAP and PGCW14 were generated by systematic deletion experiments or random mutagenesis of upstream regulatory regions. To make PAOX1 independent of methanol induction, new engineering efforts have focused on the modification of the PAOX1 core promoter using random or rational approaches, as well as transcriptional regulatory circuits. These efforts in *P. pastoris* promoter engineering have resulted in improved, sequence-diverse synthetic promoter variants that allow coordinated fine-tuning of gene expression for a wide range of biotechnological applications [183].

**Figure 6:**
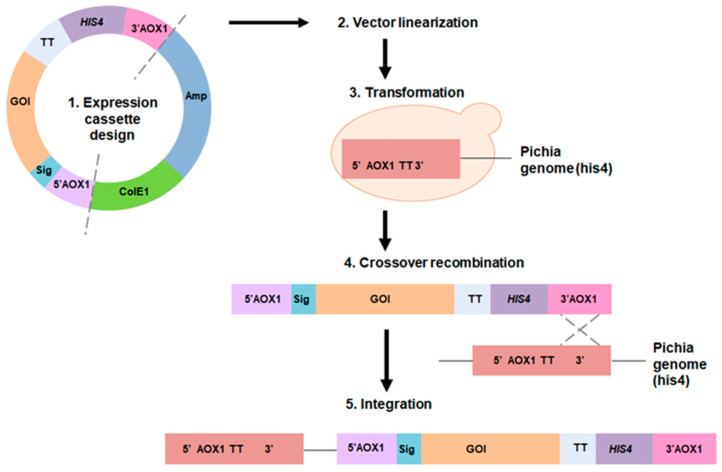
Schematic of integrating a commercial expression vector into the *P. pastoris* chromosome. Main steps for the cloning and heterologous expression of L-ASNase in *P. pastoris* cells numbered 1–5. Under step 1, the dotted line indicates unique restriction sites for Not I and Bgl II restriction enzymes. Pichia expression vectors include the common and optional features such as: (1) 5′ AOX1—including 5′HR (5′ homologous region) and promoter; (2) Sig—sequence for an N-terminal protein secretion signal (optional); (3) MCS for insertion gene of interest (GOI); (4) TT—yeast transcription termination sequence; (5) yeast marker (e.g., *HIS4*); (6) 3′ AOX1; (7) bacterial marker (e.g., Amp); (8) bacterial origin (e.g., ColE1). The AOX1 promoter and a homologous region downstream of AOX1 (3′ AOX1) allow crossing over to replace the chromosomal AOX1 locus with the expression cassette. The expression cassette is stably integrated into the genome by homologous recombination.

Some important steps (Figure 6) could ensure successful cloning and expression of heterologous proteins in *P. pastoris*: selection of promoter–terminator combinations; selection of markers (drug resistance, auxotrophy); use of vector systems for intracellular or secreted expression and complementary host strain; and selection of appropriate secretion signals. The first step in the expression of a recombinant fusion protein is to clone a target gene. Then, ligation combinations are transformed into a competent *E. coli* strain and selected. Recombinant vectors should be properly sequenced over the sites where the fused DNA fragments have been cloned to ensure cloning accuracy [170].

L-ASNase was successfully produced in *P. pastoris* under the control of the AOX1 gene promoter to overcome the low production of L-ASNase II in *S. cerevisiae*. The recombinant *P. pastoris* strain produced 800 g^−1^ of enzyme per dry cell mass, which was seven times more than that produced by *S. cerevisiae*. A volumetric yield of 85,600 U/L and a total volumetric productivity of 1083 U/L h^−1^ were then obtained from high-density cell cultures [184].

L-ASNase from *E. chrysanthemi* NCPPB1125 was expressed in *P. pastoris* strains SMD1168 and X33. The recombinant vector pPICZA was constructed by deleting the signal peptide from the gene encoding L-ASNase and transforming *E. coli* DH10B competent cells. After DNA extraction, the resulting recombinant vector, pPICZAasn, was transformed into competent *P. pastoris* SMD1168 and X33 cells. Extracellular L-ASNase activity was shown to be stable and strong in *P. pastoris* SMD1168. After purification, a specific band of approximately 45 kDa appeared, showing the glycosylated protein with a specific activity of 6.251 Umg^−1^ [185].

#### 2.3.2. Affinity Tags

Several strains of *P. pastoris* are already marketed under the name Glycoswitch^®^, which can perform both homogeneous and humanized glycosylation in recombinant proteins [186], which may be effective for covering immunogenic epitopes of bacterial proteins such as L-ASNase.

Effer et al. created two alternative vectors for the expression of L-ASNase expressed by the *E. chrysanthemi* bacterial asnB gene and investigated the influence of the His-tag on the extracellular production of L-ASNase in the Glycoswitch^®^ strain *P. pastoris*. The *P. pastoris* Glycoswitch^®^ SuperMan5 (his-) strain and the *S. cerevisiae* pJAG-s1 α-mating factor expression vector (used for extracellular secretion of recombinant proteins) were used. The synthetic asnB gene (UniProtKB—P06608) was used as a template for amplification by inserting a His-tag at the C-terminus or by removing the His-tag in the reverse primer. When the His-tag was removed, the mechanisms of cellular expression and protein release were dramatically improved. Three-dimensional protein modeling suggests that additional structures (His-tags) may have a detrimental effect on the native conformation and folding of L-ASNase, as well as on the expression and secretion of this enzyme by such cells [187].

The expression of *E. coli* eukaryotic L-ASNase in yeast was reported by Sajitha et al. The experiments were carried out in the PichiaPink^TM^ (pPink 4) expression system, which lacks both the pep4 and prb1 proteases. The ansB gene, which encodes L-ASNase in *E. coli*, was cloned and inserted into the pPink HC (high copy number) plasmid and transformed into PichiaPink (pPink 4). The *S. cerevisiae* alpha mating factor for secretion of the recombinant protein and the His-tag for easy purification were also cloned in frame with the gene. The recombinant enzyme was extracellular and had an activity of 2.5 IU/mL.

The expression of L-ASNase from *E. coli* in yeast was reported by Sajitha et al. The experiments were carried out in the PichiaPink^TM^ (pPink 4) expression system, which lacks both the pep4 and prb1 proteases. The ansB gene, which encodes L-ASNase in *E. coli*, was cloned and inserted into the pPink HC (high copy number) plasmid and transformed into PichiaPink^TM^ (pPink 4). The *S. cerevisiae* α-mating factor for secretion of the recombinant protein and the His-tag for easy purification were also cloned in frame with the gene. The recombinant enzyme was extracellular and had an activity of 2.5 IU/mL [188].

*S. cerevisiae* L-ASNase II (ScASNaseII) has great potential to replace bacterial ASNase, but its endogenous production results in a hyper-mannosylated immunogenic enzyme. The mature sequence of the protein encoded by ASP3 (amino acids 26–362) was fused to the *P. pastoris* acid phosphatase secretion signal sequence to produce the extracellularly produced recombinant ScASNaseII enzyme. As a result, L-ASNase with a specific activity of 218.2 IU mg^−1^ was expressed, and the resulting enzyme was 40% more stable when incubated with human serum than the commercially available *E. coli* ASNase (EcASNaseII). In addition, ScASNaseII has 50% less cross-reactivity with an anti-ASNase antibody raised against EcASNaseII than with ASNase from *E. chrysanthemi* [189].

#### 2.3.3. Signal Sequence and Post-Translational Modifications

Compared to the *E. coli* or *B. subtilis* systems, the *P. pastoris* expression system can fold and post-translationally modify proteins [190].

In yeast, the secretory expression of recombinant proteins requires the presence of a signal sequence that facilitates the entry of the recombinant protein into the endoplasmic reticulum, which is the first step in secretory expression. Upon entry into the endoplasmic reticulum lumen, the enzymes Kex2 and Ste13 normally cleave this signal peptide. The *S. cerevisiae* α-factor signal sequence (89 amino acids) and its truncated forms have been successfully used for secretion in *P. pastoris*. This signal peptide sequence has a single Kex2 cleavage site between Arg and Glu [173]. This peptide has been used to increase L-ASNase production in studies such as [187,188,191].

Recombinant proteins with mammalian-type glycosylation have been expressed due to *P. pastoris* strains that have developed the N-glycosylation pathway. The main aim is to knock out the endogenous glycosyltransferase gene that produces hyper-mannosylated molecules. The result is greater homogeneity of the final bioproduct, a homogeneous pattern of glycosylation, high expression of recombinant proteins, prevention of aberrant glycosylation of products and greater homogeneity of the process. In one study, a novel L-ASNase from *E. coli* was successfully expressed, secreted and glycosylated in a human-like manner using an engineered *P. pastoris* strain GS115 and *P. pastoris* SuperMan5 (his^−^). Wild-type L-ASNase and the triple mutants showed increased stability in human serum compared to non-glycosylated L-ASNase. Also present in the glycosylated forms of L-ASNases, the Man5GlcNAc2 glycans were able to reduce the immunogenicity of the enzyme in vitro [192].

A novel L-ASNase from *E. chrysanthemi* (Erwinase) was expressed in the Glycoswitch^®^SuperMan5 (his^−^) *P. pastoris* strain capable of producing proteins with human-like glycosylation. Three extracellular and glycosylated active recombinant Erwinase variants were found, two of which were tetrameric and one monomeric. When the heavier tetramer was compared with the native enzyme, the activity was lower (3.56%). The tetramer and the monomer were less dense and showed 68.5 and 11% of the original enzymatic activity, respectively. Both enzymes showed stable enzymatic behavior under physiological conditions and high catalytic efficiencies. The glycosylated Erwinase significantly reduced antibody recognition in an ELISA, suggesting that the glycans acted as a cloaking agent for the antibodies [193].

#### 2.3.4. Optimization of L-Asparaginase Production in *Pichia pastoris*

Genetic modification of strains or at the cultivation process level in bioreactors is used to address existing shortcomings in the *P. pastoris* production system. In general, bioprocess design seeks to identify optimal conditions for biomass growth and product formation, such as pH, temperature, oxygen and nutrient supply. The formulation of an appropriate way of adding carbon and energy substrates, i.e., the feed profile or feeding strategy, is critical to understanding the physiological limits of *P. pastoris*. Appropriate growth conditions and feeding techniques differ depending on whether recombinant proteins are produced under the control of the inducible AOX1 promoter or the constitutive GAP promoter in Mut+ or MutS *P. pastoris* strains. Currently available culture protocols are primarily based on those outlined in Invitrogen’s commercial expression kits. However, a recent trend is to move away from standard protocols (i.e., fixed recipes for growth-dependent and growth-independent production kinetics) toward a conceptual approach that allows the development of a specific process strategy tailored to both characteristics of specific bioreactor equipment [194] and a specific product/genetic construct combination [134].

The recombinant *P. pastoris* Mut strain was used to biosynthesize the alternative *S. cerevisiae* ASNase II encoded by the ASP3 gene using different cultivation techniques. The optimal technique for the production of ASNase II was a glycerol-fed batch phase with pseudo-exponential feeding followed by induction with continuous methanol feeding [195].

The ASP3 gene from *S. cerevisiae* was cloned and expressed in *P. pastoris* to reduce immunological responses to bacterial L-ASNase, and further SmF synthesis of the recombinant strain resulted in an enzyme yield of 800 U/g dry cell mass [184].

Selection of *P. pastoris* Glycoswitch^®^ strains and oxygen–methanol control in fed batches resulted in the increased synthesis of glycosylated L-ASNase from *E. chrysanthemi*. *P. pastoris* strains were used, three of which were glycoengineered Glycoswitch^®^ SuperMan5 strains (och1-1, GAP-mannosidaseHDEL) (Biogrammatics Inc., Carlsbad, CA, USA) and one of which was a WT strain (ATCC^®^ 28485TM) (non-glycoengineered strain). The glycoengineered strains differ in that one is auxotrophic for histidine (his^−^) and the other two are prototrophic for histidine (his^+^). Two glycoengineered strains, one his^+^ and one his^−^, were transformed with the plasmid pJAG_s1 (Biogrammatics Inc.) containing the *E. chrysanthemi* gene ansB and the *S. cerevisiae* α-mating factor secretion signal for extracellular production. In rich media, the strain auxotrophic for histidine performed worse than the prototrophic strain in terms of cell concentration and L-ASNase activity, with values of 11.9 and 14.3 gdcw/L and 9400 and 10,700 U/L, respectively. Consistent results were obtained when cultured on specific BSM media supplemented with two levels of histidine (480 and 600 mg histidine/L). In addition, auxotrophy may have impaired metabolism and influenced heterologous expression. Therefore, the prototrophic strain was chosen to avoid working with histidine supplementation in the bioreactor. Subsequently, the induction phase was improved by switching from a pulse technique to continuous methanol feeding controlled by dissolved oxygen tension (DO-stat) at 20%, resulting in a 2-fold increase in maximum volumetric activity and cell concentration [191].

## 3. Approaches for Engineering Novel L-ASNases

**Figure 7:**
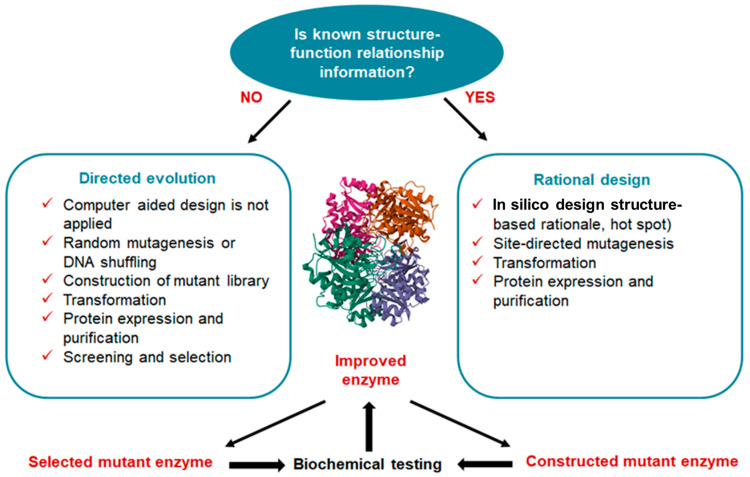
Comparison of directed evolution and rational design to the molecular modification of enzymes. Directed evolution is an approach that involves generating genetic diversity and selecting for improved variants to produce a protein with improved characteristics. In contrast to rational methods, directed evolution generates random mutations in the gene of interest and does not require any information about the protein structure. Rational design emphasizes an early understanding of protein structure and amino acid relationships. Rational design uses site-directed mutagenesis and computer modelling tools. In the center is unmodified L-ASNase from *E. coli.* (Protein Data Bank data source 6V5F. The structures were visualized using the PyMOL program; Schrodinger Inc., New York, NY, USA).

There are two typical approaches to the molecular modification of enzymes: directed evolution and rational design (Figure 7) [196]. Combinatorial techniques (e.g., directed evolution) based on random mutation have proven effective in repurposing existing proteins to take on new roles and in generating new catalytic activities from random sequences. However, because there is so much sequence space to explore in a design challenge, certain practical choices must be made to reduce the search space to a feasible size. It is therefore difficult, if not impossible, to make a sharp distinction between rational and combinatorial techniques. Understanding the molecular basis of the protein feature that is the focus of the design study (structure–function–energetics relationship) is a fundamental requirement of rational design [197].

It is essential to be able to predict the type and position of mutations that will increase enzyme activity. Recruitment and enhancement of pre-existing activity in the protein scaffold are key components of the current paradigm of enzyme evolution. Many enzymes exhibit promiscuous activity with respect to secondary substrates in addition to high catalytic efficiency with respect to their native substrate. The idea that this latent promiscuous behavior serves as a reservoir for improving the functionality of enzymes has received much attention. Much research in recent years has identified genetic changes that are associated with increased activity during evolutionary cycles. Directed evolution and bioinformatics-based methods can be used to optimize enzyme activity. While directed evolution and bioinformatical research have revealed the contributions of both proximal and distal mutations, these studies are more biased in that they represent only mutations that were selected during adaptation. Although there are several mutations that increase activity > 10 Å from the catalytic center, in general, mutations closer to the active site (<10 Å) are likely to have a greater effect. Distal mutations can also lead to significant increases in activity. The overall improvement in activity relative to the total number of mutations varies significantly depending on the enzyme model, according to a new comprehensive review of directed evolution research [198].

### 3.1. Directed Evolution for Designing L-ASNases

**Figure 8:**
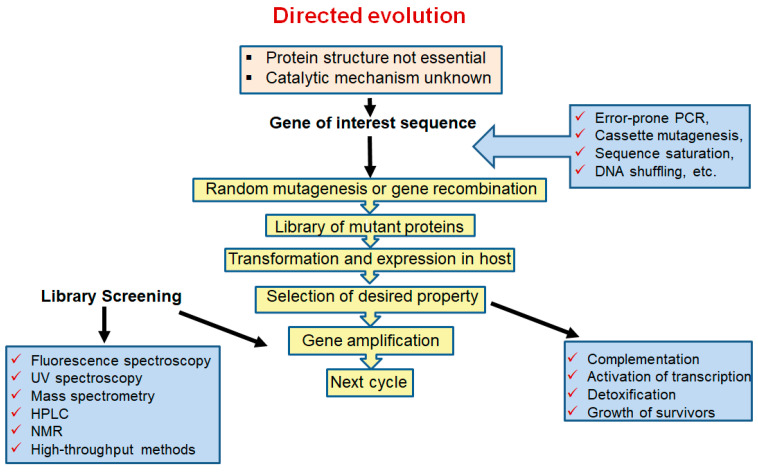
Directed evolution strategy. In vitro directed evolution experiments require the selection of (1) the genes encoding the desired enzymes; (2) a suitable expression system; (3) a successful approach for generating mutant libraries; and (4) a suitable screening or selection mechanism. There are three crucial processes involved in a directed evolution experiment: (a) generation of a mutant protein library by the mutagenesis methods; (b) screening and selection to eliminate non-functional variations. To express proteins, mutants are often transferred into bacteria or yeast hosts before being tested for functionality. Individual protein variations are screened to see if they have the desired activity; (c) amplification of the gene of interest by PCR.

The directed evolution strategy (Figure 8) was developed in the early 1990s [196]. In contrast to rational design, directed evolution allows for the relatively rapid engineering of enzymes and does not require a thorough understanding of structure-function correlations. Although on a much shorter timescale, it mimics the course of natural evolution [199]. Natural evolution is based on the principles of recombination and mutation of genetic information and fitness-based selection [200]. The discovery of PCR in 1985 enabled the application of random and saturation mutagenesis procedures. Later, directed evolution approaches improved the function and enantioselectivity of many enzymes and were widely used to tailor metabolic pathways and genomes [201].

A typical directed evolution experiment begins with the selection of a library of parent DNA sequences encoding proteins that contain some of the desired feature. The diversity of sequences being investigated is then expanded by adding random point nucleotide mutations and/or recombining DNA fragments during the mutagenesis process [202]. Mutagenesis methods (random, recombination, rational) are used to create DNA libraries by specifically targeting the gene encoding the enzyme (Table 1). Random mutagenesis is commonly used in directed evolution and has been shown to be successful in improving enzyme properties, particularly when the relationship between enzyme structure and function is unknown [199]. Random mutagenesis approaches do not target specific positions in the sequence. Recombination-based approaches differ significantly from random mutagenesis procedures in that they generate libraries of combinatorial variations by combining segments from several functional biomolecules. In general, recombination libraries have a larger percentage of functional variants, avoid the introduction of stop codons, and can lead to the detection of epistatic mutations. Rational mutagenesis focuses on changing a small number of sites in the target sequence, which must be decided on the basis of prior knowledge, as opposed to random mutagenesis techniques [203].

The cell’s biosynthetic machinery is then used to generate the relevant enzyme variants, either in vitro or in a suitable host organism. Finally, screening or selection is used to find catalysts with improved properties, and their corresponding genes are then amplified [23].

The enzymatic activity of L-ASNase I from *Bacillus megaterium* H-1 has been improved using directed evolution approaches. All five mutants showed a significant 3-fold increase in L-ASNase I enzyme activity after two cycles of error-prone polymerase chain reaction (epPCR) and two rounds of sequential DNA shuffling. The D-9B and DD-12G mutants had relatively high catalytic activity, which was 20.22 and 21.33 times higher than that of the wild-type enzyme, respectively. In addition, the catalytic efficiency (k_cat_/K_m_) of D-9B and DD-12G was increased compared to WT (3.39 min^−1^ mM^−1^) and was 132.73 min^−1^ mM^−1^ and 146.39 min^−1^ mM^−1^, respectively (1 mM^−1^). Compared to the wild type, the DD-12G mutant could tolerate a wider pH range and higher temperatures. The results also showed that fried potato chips pretreated with the DD-12G mutant could remove approximately 88.5% (0.978 mg/kg) of acrylamide [217].

Generally, L-asparagine has a millimolar K_m_ in all human L-ASNases. Recently, a low-K_m_ human L-ASNase was generated from a low-K_m_ guinea pig L-ASNase (gpASNase I) using directed evolution methods. To generate genetic variation in hASNase1, epPCR was used (on the full-length sequence and the C-truncated construct). Combinatorial active-site-saturation test (CAST) mutagenesis was used to generate eight libraries with simultaneous randomization at two or three amino acid positions. Since gpASNase1 and human L-ASNase 1 (hASNase1) share approximately 70% amino acid identity, DNA shuffling was used to construct humanized chimeras of gpASNase1 and hASNase1. Two of the clone-discovered sites, 63 N-hC and 65 N-hC, had 85.7% and 87.1% identity with hASNase1, respectively, but had a K_m_ comparable to gpASNase1. Compared to hASNase1, these clones had 100–140 times higher catalytic efficiency and retained their ability to kill leukemia cells in vitro [218].

L-ASNases from *Erwinia carotovora* and *E. chrysanthemi* were subjected to directed evolution by using SteP and site-saturation mutagenesis techniques to develop variations with increased thermostability. Increasing the stability of L-ASNase through protein engineering minimizes immunosuppressive effects by lowering the therapeutic dose. A point mutation variation (Asp133Val) was discovered after one cycle of site-saturation mutagenesis at amino acid position 133 by overlap extension PCR. It was revealed that this site is critical for the thermal stability of the enzyme [219].

A novel variant of L-ASNase lacking glutaminase activity in vitro was developed by directed evolution using genes from *E. chrysanthemi* and *Erwinia carotovora* L-ASNases. The staggered extension protocol was used to generate a library of enzyme variants, which were then tested using activity assays. One version of the *E. carotovora* enzyme was found to have no detectable glutaminase activity. Sequence analysis revealed that this variant carried the Leu71Ile point mutation. By steady-state kinetic measurements and study of the L-asparagine hydrolysis V_max_ and V_max_/K_m_ pH dependence, it was found that the mutation significantly altered the binding and catalytic properties [220].

Site saturation mutagenesis libraries using the overlap extension PCR technique have been used to generate human L-ASNase (hASNase-3) mutant libraries with increased activity. The main obstacle in this particular engineering design is to create mutations that can improve catalytic activity without affecting intramolecular processing. The 10^3^-fold smaller two-codon randomization library was able to reveal the regions of the enzyme that had a favorable effect on the catalytic properties during mutagenesis. Subsequent rounds of mutant selection led to the discovery of double mutant variants that were more catalytically efficient than the WT enzyme with respect to K_m_ and k_cat_ toward L-asparagine. For example, in terms of overall catalytic efficiency (k_cat_/K_m_), the DM1 (Ile189Thr, Val190Ile) variant showed a 6-fold improvement; k_cat_ increased 2.5-fold, and the K_M_ value decreased 2.3-fold [221].

#### High-Throughput Screening

High-throughput screening (HTS) is the rapid evaluation of hundreds to millions of samples for biological activity at the molecular, cellular or model organism level using automated equipment. Identifying compounds (called hits) with pharmacological or biological activity is a common task for HTS in the pharmaceutical and biotechnology industries. These findings serve as the basis for medicinal chemistry optimization in pharmacological research or new drug development [222]. Finding interesting but rare enzyme variations in large libraries is the main problem for every directed evolution project. To accomplish this, numerous approaches for testing the catalytic activity or binding affinity of library members have been developed. Selection techniques that link enzyme activity to cell survival are commonly used. High-throughput screening approaches are advantageous for evolutionary issues with few solutions [23].

High-throughput screening is a rapid and sophisticated laboratory technique that has been applied to many microorganisms. By identifying desired outcomes and using reverse engineering, it is possible to obtain strains with specific mutations associated with the desired phenotype. The “optimal host strain” or “optimal expression system” is often determined or selected as a function of the amount, quality and/or location of the expressed protein of interest relative to other populations of phenotypically distinct host cells in the matrix. The best host strain is one that produces the desired polypeptide according to specifications [223]. Overall, high-throughput screening accelerated the time-consuming and costly process of strain engineering by enabling massively parallel characterization of microorganisms [167].

Many studies have been carried out to find suitable alternative L-ASNases using high-throughput screening to test a variety of microorganisms, such as bacteria, fungi and yeast. High-throughput screening allows the rapid identification of new sources of L-ASNase. However, only a small number of potential L-ASNases have been identified [224].

For example, high-throughput screening of *P. pastoris* clones was used to investigate the influence of various cultivation parameters on *P. pastoris* cell growth [225]. Various expression profiles and ratios were also rapidly investigated to optimize gene co-expression in *P. pastoris* using 168 synthetic bidirectional promoters (BDPs) and naturally occurring BDPs as a repository of components [226].

FACS-based screening was performed for hASNase-3 mutant libraries. This HTS is based on the use of a 5-gene-deletion *E. coli* strain (devoid of all genes involved in L-asparagine production) whose survival is completely dependent on the availability of L-asparagine from the growth medium. Genetic complementation with hASNase-3 mutants grown in L-asparagine-free minimal medium rescues these *E. coli* cells by supplying L-asparagine generated by enzyme-catalyzed L-asparagine hydrolysis, with bacterial cell growth and proliferation proportional to the activity of the hASNase-3 mutants. Enhanced green fluorescent protein (eGFP) co-expression provided an additional level of measurement of L-asparagine availability by correlating intracellular eGFP fluorescence intensity with mutant L-ASNase activity [221].

### 3.2. Rational Design Strategies and Bioinformatic Tools to Improve the Structural and Functional Properties of L-ASNases

**Figure 9:**
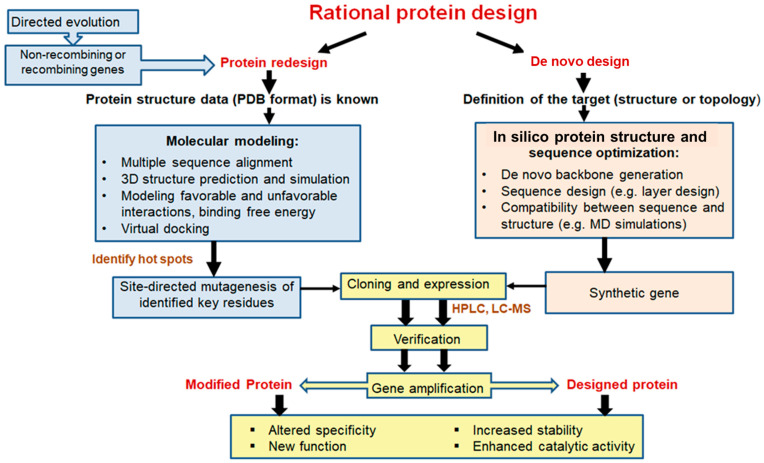
Rational design principle. This approach uses extensive knowledge of a protein’s structure and function to make desired changes to it. Proteins can be rationally designed in two ways: (1) by making calculated changes to a known protein structure and sequence (protein redesign) or (2) by generating amino acid sequences compatible with existing or assumed template backbone structures (de novo design). Techniques of rational design include (a) substrate docking, (b) molecular dynamics simulations, (c) computational molecular modeling, and (d) site-directed mutagenesis. Rational protein design techniques generate protein amino acid sequences that are predicted to fold into specific 3D structures. These predicted sequences should then be experimentally confirmed by chemical synthesis of an artificial gene, followed by protein expression and purification.

Bioinformatic techniques can be used to increase enzyme activity, stability and substrate specificity. Rational design involves extensive knowledge of enzyme structure and catalytic mechanism, as well as information on evolutionary linkages, functional sites, and mutations [196]. Rational design engineering is a computational approach to protein modification (Figure 9). Proteins are inherently unable to withstand harsh industrial environments, such as elevated temperatures, high pH and high salt concentrations, as they have evolved to function under mild conditions. To improve their industrial potential, many in silico approaches have been developed to estimate the effect of mutations on protein stability. The rational design approach requires a crystal structure data set, biophysical data, protein function and sequence-based data, in particular consensus sequences that are advantageous for protein folding during natural evolution [227]. The rational approach requires a thorough understanding of the structural elements that make up the active site of an enzyme and how they affect the function of the enzyme and focuses on understanding the relationships between enzyme structure and function to predict likely mutants with desired properties and introduce mutations through site-directed mutagenesis [196].

Large virtual libraries of enzyme variants are generated in silico by computational engineering. Only a small number of hits (tens to several hundred) are carefully screened and tested in the laboratory after the designs have been automatically ranked and scored, e.g., by energy scoring functions or geometric constraints. A variety of technologies are used to construct libraries, many of which introduce many mutations simultaneously [197].

Enzyme kinetic parameters, substrate selectivity, thermostability and tolerance to organic solvents can all be improved through rational design engineering. In addition, the size of the library can be significantly reduced, requiring less time and effort [227]. The structure of the enzyme, multiple sequence alignment and computational methods are the three main strategies used to pinpoint the exact residues to change [23]. Rational design is a promising strategy to improve the enzymatic performance of L-ASNase [16].

#### 3.2.1. Multiple Sequence Alignment

In the absence of structural knowledge of the target protein, the simplest approach is to perform sequence analysis by multiple sequence alignment (MSA). Using MSA, the sequence of the target protein can be aligned with other similar homologous proteins from other sources. Such an alignment provides information about amino acid residues that are conserved across sequences and are critical for protein function and stability. These residues are hotspots in the protein that can be used as mutation targets [228]. MSA has also been used to improve computational enzyme design through a maximum entropy strategy, which can improve enzyme efficiency and stability [229].

MSA constructs two phylogenetic trees of two interacting proteins, and the discovery of strong correlations between phylogenetic trees is used to infer likely coevolution and interactions [230]. MSA approaches include progressive alignment, iterative alignment, consistency-based alignment and profile-based alignment. Progressive alignment is a popular heuristic that creates a final MSA by integrating pairwise alignments, starting with the most similar pair. The Clustal family is the most widely used implementation of progressive alignment; the most prominent techniques are ClustalW and ClustalOmega. Iterative algorithms, such as MUSCLE, MAFFT and ProbCons, overcome the greediness of the progressive alignment method through an alignment refinement process that optimizes the achieved output. Evolutionary and genetic algorithms are extensions of iterative algorithms that use a stochastic process to improve the final answer. Consistency-based approaches improve the outcome by using consistency information from different pairwise alignments. T-Coffee is the most common approach in this category [231].

Analysis of the proteome of 176 archaea using the ClustalOmega and T-Coffee tools revealed two new families of archaeal asparaginases: Asp2like1 and Asp2like2 [232]. Conservative sequences for the most common asparaginases *E. coli*, *E. chrysanthemi*, *E. carotovora*, *Helicobacter pylori*, *Rhodospirillum rubrum* and *Wolinella succinogenes* were identified using the PRALINE and CLUSTAL instruments [233].

The two most popular phylogenetic methods for stability engineering are ancestral sequence reconstruction (ASR) and consensus design. ASR uses a combination of phylogenetics, evolutionary theory, synthetic biology and protein biochemistry to infer the sequences of ancestral proteins and then characterize them in the laboratory. Two properties of reconstructed ancestral proteins/enzymes are commonly reported—high thermostability and high catalytic activity—compared with their contemporaries [234]. The inference of ancestral sequences can be performed with three different approaches: maximum parsimony (MP), maximum likelihood (ML) or Bayesian (often called hierarchical Bayesian (HB)) methods, the latter two being the so-called probabilistic methods. All three require MSA of the proteins of interest and a phylogenetic tree explaining relationships between the orthologues selected, and ML and HB methods allow the use of different models of evolution (often called substitution models) [235].

Consensus designs were created to find changes in the conserved positions of alignments and restore such changes to the consensus state [236]. The consensus residue is stabilized at each position in the protein family. As a result, stable enzymes can be produced while introducing mutations at consensus sites. This increased the specificity of *E. chrysanthemi* L-ASNase. Conservative sites were found in the active center of this enzyme, leading to the production of an active mutant with 25-fold lower glutaminase activity [237].

Protein structure prediction and identification of functional regions can be performed using coevolutionary analysis. This approach to the rational design of enzymes was made possible by the experimental confirmation of the identified coevolving residues. Carrying out MSA, calculating coevolutionary indicators and experimental verification are frequent steps in coevolutionary analysis algorithms [238]. MSA and coevolutionary analysis are routinely used to extract information from target protein sequences. The activity of the protein is closely tied to preserving the three-dimensional structure, so structure-based design is more efficient in discovering critical L-ASNase residues [239].

Safari et al. examined a novel halo-thermotolerant strain of *Bacillus* sp. SL-1 as a source of novel L-ASNases by ClustalW and Jalview. *Bacillus* sp. SL-1 provided two L-ASNase genes, ansA1 and ansA3. Catalytic activity experiments on the discovered L-ASNases (ansA1 and ansA3) from *Bacillus* sp. SL-1 revealed that only the ansA1 gene encodes an active and stable homolog (L-ASNase A1) with significant substrate affinity toward L-asparagine. The isolated ASPase A1 enzyme had k_cat_ and K_m_ values of 23.96 s^−1^ and 10.66 µM, respectively. The k_cat_ value of ASPase A1 was higher than the calculated value for *E. coli* therapeutic L-ASNase. The K_m_ value of L-ASNase A1 was approximately 43 and 67 times lower than that of *B. subtilis* B1106, respectively, while it was close to that of *B. licheniformis* RAM-8 L-ASNase. In addition, no L-glutaminase activity was detected in the newly active enzyme (A1) [240].

A new L-ASNase from the actinomycete *Mycobacterium gordonae* (GmASNase) was identified in a study by Chi et al. by multiple nucleotide sequence alignment, which showed that GmASNase had the highest similarity to some mycobacterial L-ASNases. *E. coli* BL21 (DE3) was then used to clone and express GmASNase. At pH 9.0 and 50 °C, the purified GmASNase exhibited a maximum specific activity of 486.65 IU mg^−1^. In addition, GmASNase was active below 40 °C and stable over a wide pH range of 5.0–11.0. With K_m_, k_cat_ and k_cat_/K_m_ values of 6.025 mM, 11,864.71 min^−1^ and 1969.25 mM^−1^ min^−1^, respectively, GmASNase also showed good substrate specificity for L-asparagine and poor affinity for D-asparagine (22%). After GmASNase treatment of potato chips prior to frying, the acrylamide content decreased by 65.09% compared to the untreated control [241].

MSA was used to search for homologs of L-ASNases from the thermophilic bacterium *Melioribacter roseus* (*M. roseus*). Two enzymes with L-ASNase activity were found in *M. roseus* P3M-2, WP_014855981.1 and WP_014855710.1, encoded by the genes MROS_RS06765 and MROS_RS05340, respectively. Multiple sequence alignment in Clustal version 2.1 was performed using bacterial type II L-ASNases from *E. coli* (AAA23445.1), *P. atrosepticum* (AAP92666.3), *R. rubrum* (QXG80441.1) and *D. chrysanthemi* (AAS67028.1) and homologs from the Bacteroidetes-Chlorobi group. A homology search revealed that the putative isoaspartyl peptidase/L-ASNase WP_014855981.1 had the highest identity with the previously identified *E. coli* isoaspartyl peptidase/L-ASNase WP_146849425.1, with 55.2% classified as a plant-type EcAIII L-ASNase. The second L-ASNase found in the *M. roseus* P3M-2 WP_014855710.1 genome was characterized as a type II (class 1) bacterial L-ASNase (MrAII). Amino acid sequence comparison revealed that MrAII shares 78.7–76.1% similarity with a number of uncharacterized L-ASNases from recently annotated genomes of the bacterium Ignavibacteria. The L-ASNase MrAII was selected from the two annotated in the *M. roseus* genome for further detailed elucidation [242].

A similar approach was used to find homologs of a novel L-ASNase from the hyper-thermophilic archaeon *Thermococcus sibiricus* (TsA). Amino acid sequence comparison revealed that TsA shares 77% homology with archaeal L-ASNases from *Thermococcus litoralis* (GenBank accession number WP_004066133) and 62% homology with L-ASNases from Thermococcus zilligii (GenBank accession number WP_010478656) and *Thermococcus gammatolerans* (GenBank accession number WP_01585 9055) at 61% and the well-characterized L-ASNase from *Thermococcus kodakarensis* (WP_011250607) at 63% [107].

#### 3.2.2. Homology Modeling

Three-dimensional models constructed from amino acid sequences using computer algorithms can be used to predict the structure of a protein. These approaches are based on the fact that protein structures have evolved to be more conserved than their sequences, suggesting that proteins with different sequences may have comparable folds. Homology modeling is an important and preferred method of protein structure prediction. This method is used when a template structure with a good extent of sequence similarity with the target protein sequence is available. This method assumes that protein structures are more conserved than protein sequences. Hence, an accurate model of a protein could be designed using core modeling, loop modeling, and side chain modeling followed by energy minimization. A template is searched using different sequence similarity or alignment-based search tools, such as BLAST and PSI-BLAST, and then looking for their known structures in the PDB database [243]. Many homology modeling methods/servers have been developed in recent years, including SWISS-MODEL, MODELLER, I-TASSER, ReformAlign, PyMOD, TIP-STRUCTFAST, COMPASS, 3D-PSSM, SAMT02, SAMT99, 3DPSSM, HHPRED, FAGUE, 3D-JIGSAW, META-PP and ROBETTA [228].

The most popular online tool for predicting protein 3D structure is the SWISS-MODEL server (http://swissmodel.ExPASy.org/ (accessed on 11 October 2023)). The SWISS-MODEL server can generate and improve the structural match between the target and template by automatically selecting numerous matching templates. This method was used to generate a model of *B. subtilis* strain 168 L-ASNase [244] and predicting the 3D structure of L-ASNase of *Pyrococcus yayanosii* CH1, *Thermococcus gammatolerans*, *E. coli* [16], *Cobetia amphilecti* AMI6 [245], *Aspergillus terreus* [246] and many others.

The I-TASSER service has a reference scoring system to assess the quality of the model and is intended to predict full-length 3D protein structures [247]. This program was used to predict the increased structural stability and decreased toxicity of *E. coli* L-ASNase.

MODELLER is a command line tool for comparative modeling. It constructs models using homology modeling and is modular. It is accessible both locally and via a web server [228].

Phyre2 is a protein structure, function and mutation prediction and analysis toolkit. It builds 3D models, predicts ligand-binding sites, and examines the influence of amino acid changes on protein sequences using distant homology detection methods [248]. The L-ASNase monomer structure of *Citrobacter freundii* 1101, *D. dadanii* DSM 4610, *E. coli* BL21, and *Klebsiella pneumoniae* ATCC 10031 was predicted using this program [249].

Using 3D structure prediction programs, Long et al. showed that the amino acid residues close to the catalytic cavity of the L-ASNase II tetramer from *B. subtilis* B11-06 (BsAII) contribute significantly to its catalytic efficiency and thermostability. Site-directed mutagenesis was used to generate L-ASNase mutants with improved properties. Residues G107, T109 and S166 close to the catalytic cavity were selected and substituted with Asp, Gln/Ser and Ala, respectively, to create the G107D, T109Q, T109S and S166A mutants. A structural model of BsAII was created using the SWISS-MODEL workspace and the I-TASSER server, and the PYMOL program was used to analyze the interaction of hydrogen bonds between residues. Discovery Studio 2.5 was used to analyze the surface electrostatic potential and the structure of the substrate binding pocket of the enzyme. The BsAII G107D mutant (G107Dansz) outperformed the wild-type enzyme for L-asparagine in terms of heat tolerance and activity. A comparison of G107Dansz and BsAII revealed that the substitution of L-asparagine for G107 close to the catalytic cavity resulted in minor conformational changes and a redistribution of the surface electrostatic potential, which contributed to increased protein stability and catalytic efficiency [250].

#### 3.2.3. Molecular Dynamics

Molecular dynamics (MD) simulation is the most widely used computer tool for the study of the structure and dynamic behavior of proteins to determine the molecular basis of their function. Modern force fields are becoming increasingly accurate in providing an acceptable physical description for this purpose, allowing us to mimic complicated biological systems under very realistic conditions [251]. Proteins have complex dynamics that range from picosecond to millisecond timeframes in various systems. As a result, the accessible experimental structure of the original model may not reflect the expected physiologically active state [252]. The most popular computational approaches for analyzing the molecular dynamics of proteins include GROMACS, NAMD, AMBER, CHARMM, Desmond, and OpenMM, which use force fields to simulate the dynamics of proteins [236].

A novel L-ASNase isolated from the bacterial endophyte *Bacillus stratosphericus* was further investigated using in silico and molecular dynamics simulations. Since the endophyte lacked the crystal structure of the L-ASNase enzyme, a homology model was created using MODELLER 9.15v. MDs were performed using GROMACS 4.6.5v to investigate the stability of the enzyme-substrate docking complexes. The binding affinity and molecular interactions of the enzyme L-ASNase with substrates (L-asparagine and L-glutamine) were docked using iGemdock 2.1v. As a result, binding to L-asparagine requires less energy (−71.6 kcal/mol) than binding to L-glutamine (−67.7 kcal/mol). The complex with asparagine is significantly more stable than the complex with glutamine [253].

*P. furiosus* L-ASNase mutants with improved substrate affinity and antitumor activity were modeled using the AMBER 11 program [254].

#### 3.2.4. Molecular Docking of Ligand Binding

Molecular docking, the primary approach used in structure-based virtual screening, can predict the binding mechanism and affinity of a ligand (typically a small organic molecule) within the binding pocket of the target of interest. Several docking tools and programs, such as AutoDock,17 AutoDock Vina,18 LeDock, rDock, UCSF DOCK, LigandFit, Glide, GOLD, MOE Dock, and Surflex-Dock26, have been developed for commercial and academic use over the last two decades. The sampling algorithm and scoring function, which determine the sampling and scoring power of a docking program, are the two most important components [255].

Several studies have investigated how L-ASNase binds in complex with its amino acid substrates through molecular docking, dynamics and modeling. To circumvent the toxicity associated with current L-ASNase preparations, it is necessary to identify a new serologically distinct enzyme with the same therapeutic effect. Molecular docking and molecular dynamics modeling of *E. coli* L-ASNase were performed by Erva et al. to investigate its role in the deamination of asparagine and glutamine residues. Molecular docking was performed using Hex 8.0.0, PatchDock and FireDock and other binding cavity docking tools. As a result, key residues in the binding and ligand-binding mechanisms for enzyme complexes in the apo state were identified and associated with the ligand [256].

In another study, the structural and functional properties of Erwinaze^®^ and its ligands were determined by rational design to better understand the mechanism of action of this enzyme. Using the in-house docking tool GEMDOC, molecular docking simulations of the complexes of the apo state of the enzyme and the bound state of the ligand were performed to evaluate various ligand-receptor interactions and binding free energies. MD modeling of the docked complex with the substrate ligand L-Gln revealed critical residues in L-Asn binding—Thr 15, Thr 26, Thr 27 and Met 121 and Tyr 165, Thr 167 and Asn 180 in L-Gln binding [257].

The thermal stability of L-ASNase from the hot spring strain *B. subtilis* ETMC-2 was investigated in a study by Agrawal et al. The *B. subtilis* ETMC-2 L-ASNase gene sequence was used to generate an in silico model of the L-ASNase structure. Molecular docking using AutoDock 4.2 revealed a conserved motif represented by active site residues Thr89, Thr121 and Asp122. The protein had a high degree of thermal stability, as indicated by the calculated aliphatic index (85.44) and the GRAVY value of −0.217. These values were close to those of the cloned L-ASNase. Thus, in silico research supported the properties of the cloned L-ASNase [33].

The immunogenicity and other properties of new L-ASNases isolated from Isfahan wastewater were evaluated using molecular docking and molecular dynamics modeling. AutoDock 4.2 and AutoDock Vina software were used to perform molecular docking between L-ASNase and the L-asparagine substrate. GROMACS was used for molecular dynamics experiments. Among the five L-ASNase-producing bacteria identified from *Stenotrophomonas maltophilia*, *Chryseobacterium* sp. *Chryseobacterium indologenes*, *Bacillus velezensis* and *Bacillus safensis*, *B. velezensis* outperforms *B. safensis*. The predicted binding energies of the docking complexes are −4.34 and −4.9 kcal/mol. The interaction of L-ASNase with its substrate was determined by molecular docking. Key residues in the protein-ligand complex were identified as Thr36, Tyr50, Ala47, Thr116, Asp117, Met142, Thr193 and Thr192. Molecular docking analyses also showed that L-ASNase does not lose stability, secondary structure or compactness when it binds to L-asparagine [258].

#### 3.2.5. Algorithms for Predicting Protein Stability

For the prediction of advantageous single and multiple point mutation sites to lower the Gibbs free energy of proteins, at least 22 independent computational algorithms have been reported. The main tools include the FoldX algorithm, PoPMuSiC, the iRDP web server, and CUPSAT. These calculation tools are structure- or sequence-dependent and use machine learning algorithms or energy calculation routines. Databases that record changes in protein stability (e.g., Gibbs free energy changes and melting temperatures) are also available, such as ProTherm [259].

PoPMuSiC (prediction of protein mutant stability changes) is a web service that predicts changes in protein thermodynamic stability caused by single site mutations and generates a report listing the most stabilizing and destabilizing mutations [260]. This program, in combination with others, led to the development of a thermostable L-ASNase from *Bacillus licheniformis*. PoPMuSiC enabled the identification of important amino acid residues and the improvement in the target protein’s thermal stability [261].

A rapid evaluation of the effects of mutations on the stability, folding and dynamics of proteins and nucleic acids is provided by the empirical force field known as FoldX. Based on the three-dimensional structure of a macromolecule, the program determines its free energy [262]. FoldX5 was used to mimic the effect of amino acid changes on protein free energy to produce a stable L-ASNase [16]. A novel L-ASNase with the expected improved enzymatic properties of *E. coli* L-ASNase was generated in silico using techniques involving sequence and functional analysis of known mutations of this L-ASNase. Based on 21 experimentally registered ASNase mutations, a virtual library of modified enzymes was constructed containing all 7546 possible combinations with up to 4 mutations. Using Fold X (Version 4), three-dimensional models of the proposed mutant enzymes were generated, and the stability of their folding was determined in silico. The most stable mutant, produced in cells from two different *E. coli* strains (Origami B(DE3) and BL21(DE3)pLysS), carried the Y176F/S241C double mutation. The results show that the glutaminase activity for the new L-ASNase is significantly lower (13.3 and 53.3 IU for the new enzyme and commercial enzyme, respectively) at enzyme concentrations that result in almost equal L-ASNase activity (95.4 and 83.9 IU for the new enzyme and commercial enzyme, respectively) [263].

#### 3.2.6. De Novo Computational Design

De novo protein design allows the precise construction of protein structures with exceptional stability and a wide range of configurations, not necessarily limited to those found in nature. Traditional protein engineering strategies, prior to the advent of de novo protein design, relied solely on modifying existing proteins that already had a function similar to the desired one or at least the appropriate geometry and stability to withstand the mutations required to enable the desired functions. Computational de novo protein design, on the other hand, removes this barrier entirely, providing access to a potentially unlimited number of protein forms that can be viable candidates for constructing functionality [264]. The main goals of computational protein design are the development of new proteins using data based on crystal structures, the design of de novo amino acid sequences capable of adopting a stable three-dimensional structure, and modification of existing amino acid sequences to promote protein stability [265]. Many computer-based simulation techniques have been developed to achieve this, ranging from changing the function of the active site to creating a new catalyst. DEZYMER, ORBIT, Rosetta, SABRE, Pocket Optimizer, DeepEC, Enzyme Miner and EvoDesign are just a few [236]. Generally, the de novo protein design process consists of four sequential steps: (a) defining the target, which is usually a structure or topology, (b) generating suitable protein backbones, (c) designing matching amino acid sequences, and (d) evaluating the compatibility of the designed sequence with the target structure [264].

The development of de novo enzymes usually begins with the design of a theoretical active site (theozyme) with the right functional groups capable of catalyzing the reaction of interest. The second part of the process involves searching a database of protein structures for a protein scaffold. This is the limiting stage of the process, as it is difficult to achieve a perfect match between the newly generated active site and the existing protein scaffold. Once the scaffold and catalytic residues have been selected, they are ranked in optimal positions for the catalytic process. The best variants are selected for in vitro expression and experimental testing after being ranked according to desired properties such as affinity, catalytic rate and stability. In most cases, the initial designs do not exhibit the required catalytic rates, requiring either in silico refinement or multiple rounds of directed evolution [266].

To remove T-cell epitopes from *E. coli* II L-ASNase, computer structural design of the protein and epitope prediction was performed in the Rosetta program while maintaining native enzymatic activity and stability. The Rosetta design reduces the amount of expected MHC-binding peptides while increasing the energy of the protein in the epitope regions. Two of the five mutations adopted residue identities observed in the sequences of experimentally isolated activity-preserving variants after Rosetta redesign for all 24 epitope residues, with 1 mutation occurring at a site not investigated earlier. The combined strategy allowed the exclusion of epitope-like regions in L-ASNase while maintaining native values for a number of expected protein stability indicators [267].

#### 3.2.7. Machine Learning Approaches

Machine learning techniques are primarily intended for postprocessing rescoring and the training takes place using experimental datasets employing a variety of algorithms.

AlphaFold2, a protein structure prediction program based on machine learning techniques, was released by DeepMind in 2021 [64]. In the absence of an experimental structural model for the rhizobial L-ASNase (ReAIV) enzyme, Zielezinski et al. used AlphaFold2 and Rosetta to estimate its structure. The Rosetta method and AlphaFold2 predicted the structure of the ReAI. Based on a global pairwise sequence alignment between the prototype of the enzyme and all its orthologous sequences from different bacterial species, the degree of sequence identity mapped to the structures of the L-ASNase protein (EcAI, EcAII, EcAIII, ReAIV and ReAV) was evaluated. As a result, the structure of the novel L-ASNase ReAIV was discovered and characterized using machine learning [59].

Machine learning techniques such as artificial neural networks (ANNs) and genetic algorithms have been found to replicate certain features of biological information processing for data modeling and could be effective in media optimization. For the production of *Enterobacter aerogenes* MTCC 2823 L-ASNase in submerged fermentation, fermentation media components, including carbon and nitrogen sources, were optimized using a genetic algorithm combined with an artificial neural network algorithm. With a maximum predicted L-ASNase activity of 18.59 IU/mL, the ANN-based genetic algorithm indicated the ideal concentration of media components to be 2.09% sodium citrate, 0.25% DAHP (3-deoxy-D-arabinoheptulosonate 7-phosphate) and 0.92% L-asparagine. Under the predicted ideal conditions, an experimental L-ASNase activity of 18.72 IU/mL was achieved [268].

## 4. Combining Computer Design Techniques to Obtain L-Asparaginases with Improved Properties

### 4.1. Modifications to Increase the Expression

Recent research indicates that rare codons are critical for protein activity and folding. However, substitution of these rare codons can lead to protein expression problems due to premature translation termination and pausing. Protein expression problems may be resolved by paying close attention to ribosomal pausing codons. Mortazavi et al. studied enzyme properties such as CUP (codon usage preference) and a rare codon cluster (RCC) in L-ASNase II Pfam PF00710 in silico. By modeling the L-ASNase 3D structure, some rare codons were discovered that may play a crucial role in the structural and functional properties of the enzyme. It is well known that changes in CUP lead to a number of problems caused by heterologous gene expression. The characteristics of the substrate-binding sites were then investigated using the molecular docking approach. AutoDock Vina was used to determine the substrate-binding sites. Several amino acids with structural similarities to the substrate-binding site of L-ASNase (2jk0) were identified. The Swiss Model and I-TASSER web server were used to model the 3D structure of the L-ASNase II enzyme. The Sherlock program was used to study its rare codons. GCUA tools (http://gcua.schoedl.de/seqoverall_v2.html (accessed on 11 October 2023)) were used to investigate the diversity in CUP of the L-ASNase II gene. Five unusual rare codons, Lys195, Leu30, Lys184, Lys160 and Lys174, were found and examined in the structure of L-ASNase. Substrate binding studies revealed that Asp6, Asp77 and Thr78 bind to the active site of the enzyme. The results of this work may contribute to a better understanding of the folding and expression of L-ASNase [269].

### 4.2. Modifications to Decrease Glutaminase Activity

The dual activity of L-ASNase and L-glutaminase in bacterial L-ASNases makes therapeutic use difficult. As L-asparagine is found at approximately 50 μM in human blood, therapeutic L-ASNase must have a low micromolar substrate affinity. A low K_m_ coincides with a high k_cat_, ensuring that therapeutic L-ASNase effectively reduces endogenous L-asparagine at safe doses [270]. The percentage of glutaminase activity associated with L-ASNase should also be low, with excellent enzyme stability and half-life to make it an ideal therapeutic formulation [271].

Understanding the key features of L-ASNase substrate selectivity is a necessary step toward the development of less toxic enzyme variants. Several ways to reduce L-ASNase toxicity have been investigated, including mutagenic modification of the protein structure, generation of mutants with reduced ability to hydrolyze L-glutamine, chemical modification of specific amino acids, and changes in drug formulations [272]. An in-depth knowledge of the molecular basis controlling the selectivity between L-asparagine and L-glutamine will greatly benefit the rational design of L-ASNase variants with reduced L-glutaminase activity [109]. To achieve the reduction of L-glutaminase, a combination of computational protein engineering tools was used.

Offman et al. used a rational design to produce *E. coli* L-ASNase with enhanced anticancer activity. The effect of mutations and the cap loop on the anticancer activity of the enzyme was investigated using a genetic algorithm and molecular dynamics. A genetic algorithm was used to sample and select mutants. Molecular dynamics modeling of the wild-type complex produced a set of initial conformations for interface residues, which were selected using the genetic algorithm. A subset of the 24,000 resulting mutant complexes was selected for further study. In Amber Suite version 10, the four mutations in the genetic algorithm sample with the highest scores were selected to explore the dynamic and structural properties of the proteins. To better understand the effect of these mutations on the lid-loop stability and active site conformation, MD modeling was performed on WT L-ASNase and five mutant proteins with changes at position 24 (N24G, N24A, N24T, N24H and N24S D281E). The activity of the L-ASNases was then evaluated. The most successful drug in vitro was the N24A mutant with stable glutaminase activity. The N24A R195S mutant had comparable asparaginase activity and cytotoxicity but half the glutaminase activity of the wild type. The N24A Y250L mutant showed significantly less cytotoxicity than WT and lacked glutaminase activity. Depending on the side effects observed, two mutants were obtained: either to reduce the dose (N24A) or to reduce glutaminase activity (N24A R195S) [66].

The crystal structure of *E. chrysantemi* L-ASNase in combination with glutamine was revealed and compared with the previously published complex of *E. chrysantemi* L-ASNase with asparagine by Nguyen et al. They concluded that to design variations that discriminate against glutamine, it is necessary to introduce mutations that are incompatible with the closed state of the enzyme when binding L-glutamine but remain compatible with the closed state when binding L-asparagine [109]. Then, rational design was used to obtain new mutant variants of *E. chrysanthemi* L-ASNase with reduced L-glutaminase activity. The main strategy was to search for non-conserved residues surrounding the active site that do not necessarily interact with the substrate. Based on MSA and crystal structure analysis of ErA in complex with Asp and Glu products, amino acid residues Ala-31, Glu-63, Pro-123 and Ser-254 were identified as four potential candidate sites for mutagenesis. The ErA triple mutant A31I/E63Q/S254Q (ErA-TM2) showed the optimal combination of high L-asparaginase/low L-glutaminase properties. When the l-glutaminase activity of ErA-TM2 was examined, a dramatic change in its activity profile became apparent. ErA-TM2, a derivative of ErA-WT, a somewhat active L-glutaminase, was transformed into an enzyme with extremely low L-glutaminase activity. Its rate was reduced more than 1000-fold at relevant glutamine concentrations. In vitro, an ultralow ErA-TM2 mutant effectively killed human T-ALL LOUCY cells with fewer side effects [117].

The glutaminase-deficient mutant *E. coli* L-ASNase was efficiently designed using molecular dynamics modeling and site-saturation mutagenesis. Molecular dynamics simulations of clinically standard *E. coli* L-ASNase were used to predict the structures of mutant forms that retain asparagine but not glutamine activity. Modeling was performed in the NPT ensemble using the NAMD2 package with CHARMM force field parameters and grid-based CMAP correction. Residues that reacted preferentially with glutamine rather than asparagine but were not essential for enzymatic conversion were selected as candidates for saturation mutagenesis. The best candidate was amino acid Q59. The mutant (Q59L) showed the lowest percentage of original glutaminase activity but retained >60% of wild-type (WT) asparaginase activity. The authors also found that Q59L exhibited selective anticancer activity against L-ASNase-negative leukemia cell lines, while the WT enzyme exhibited L-ASNase-independent toxicity [103].

A new *E. coli* L-ASNase II mutant with reduced glutaminase activity was created in silico using molecular docking, molecular dynamics modeling and quantum mechanics molecular dynamics simulations. GROMACS 5.1 with the AMBER 99SB force field was used for all MD calculations for WT and mutants. Residues with a low free energy of binding to L-asparagine and a high free energy of binding to L-glutamine were selected for mutagenesis. The best binding free energy predicted by AutoDock Vina for each position was selected to investigate and analyze the mutations of interest. Mutants with a higher free energy of L-glutamine binding were then selected, and the best mutant found in this study was V27T. The V27T mutation reduced the glutamine-binding energy from 20.763 to 1.360 kcal/mol, and the RMSD value of the V27T mutant was lower than that of WT L-ASNase, indicating increased stability and rigidity of the mutant [273].

Durst et al. used site-directed mutagenesis and rational design to specifically reduce glutaminase activity in *E. coli* L-ASNase. When the crystal structure of *E. coli* L-ASNase was studied, it was discovered that the Asn248 residue, which is not part of the catalytic centers, can assist in substrate coordination in the enzyme pocket via hydrogen bonding. The AMBER force field was used to calculate the putative structural effects of mutations at position 248, the active site of many variations. Asparagine 248 was then replaced by other minimum energy residues. Molecular modeling showed that N248 is important for stabilizing both the ground and transition states of catalysis and is involved in hydrogen bonding between different subunits. All mutations at position 248 destabilized the enzyme to a similar extent. In the pH range of 5 to 8, k_cat_ was almost pH independent for all these enzymes, with a decrease in K_m_ at low pH, which was not found for the wild-type enzyme or other mutants. In addition, the N248A mutation reduced the glutaminase activity of L-ASNase in *E. coli* by up to 0.2% of the wild-type value. Meanwhile, the N248A mutant lost more than 90% of its L-asparagine activity. Given the amount of enzyme commonly used to treat ALL, 10,000 U per single dose, this ratio of activity should still result in asparagine depletion without causing a significant decrease in the much higher serum glutamine concentration [274].

L-ASNase from *Pectobacterium carotovorum* was engineered in silico to minimize glutaminase side activity for efficient treatment of ALL. In silico mutagenesis was used to generate enzyme variants with reduced glutaminase activity by changing amino acids near the ligand-binding region. Replacing the active site amino acid of the Asp96 enzyme with alanine reduced glutaminase activity by 30% while increasing asparaginase activity by 40%. Docking experiments were carried out using AutoDock 4.0, and it was found that the binding energy of the native enzyme when docked with glutamine is −8.08 kcal/mol, but the binding energy of the mutant protein is −5.97 kcal/mol. Replacing the active site with amino acids other than alanine had no effect on the activity of either asparaginase or glutaminase. As a result, an enzyme model with reduced glutaminase activity was created [275].

The effect of the N248S mutation on L-ASNase activity has been studied by Aghaeepoor et al. Molecular dynamics simulations, molecular docking and quantum mechanical/molecular mechanics studies are just some of the in silico analyses that have been carried out. The AutoDock Vina program was used for molecular docking of the substrates asparagine and glutamine in the active site of mutant L-ASNase. The N248S mutation was associated with strong L-ASNase activity, whereas glutaminase activity was decreased, according to the in silico analysis and experimental evidence [276].

### 4.3. Modifications to Increase Catalytic Activity and Specificity

An in silico method was developed to predict the kinetics (K_m_ value) of L-ASNase from an enzyme sequence and to test for the optimal alternative L-ASNase II against ALL. The *E. coli* asnB sequence was utilized to look for homologous proteins in different bacterial and archaeal phyla. This method uses sequence-based phylogenetic analysis to narrow down a small number of candidates on which virtual docking can be utilized to identify a set of optimal enzymes that may outperform commercially available enzymes. Mega-X software (https://www.megasoftware.net/) was used to create a maximum likelihood phylogenetic tree. In terms of immunogenicity, the sequences most distant from *E. coli* and *Erwinia* species were regarded as the best candidates and were chosen for further processing. The architectures of these proteins were created using homology modeling in MODELLER 9.22. Following model validation, PyMol was used to discover active sites for each protein, and asparagine was docked with these proteins to compute binding (AutoDock Vina coupled with PyRxsoftware). As a result, three potentially promising L-ASNase II enzymes produced by three Streptomyces species were predicted. They have the highest binding energy (−5.3 kcal/mol, −5.2 kcal/mol, and −5.3 kcal/mol, respectively; greater than *E. coli* and *Erwinia* L-ASNase) and were predicted to have the lowest K_ms_ since binding energy and K_m_ have an inverse relationship [277].

Zhou et al. used a semi-rational design to increase the catalytic activity of *Bacillus licheniformis* L-ASNase in silico. The generation of mutation sites was performed in COACH, and Hotspot Wizard 3.0 and EVcoupling are general servers for predicting possible functional sites. The COACH server was used to predict protein-ligand binding sites, Hotspot Wizard 3.0 was used to automatically identify hotspots, and the EVcoupling server was used to generate pairs of evolutionary binding residues. The JackHMMER method was used to filter active site residues by structural analysis and sequence comparison. This allowed the development of libraries that could make modifications directly at specific sites while reducing the screening burden. After saturation mutation, all mutants were validated for catalytic activity. As a result, five single point mutants (E102I, V104L, A180R, T223A and T325C) had relatively high enzyme activity. Based on the results of the single mutants, double, triple, quadruple and quintuple mutants were designed, as combinatorial mutagenesis allows the investigation of potential additive or even synergistic effects. After screening, a quintuple mutant, ILRAC (E102I/V104L/A180R/T223A/T325C), with significantly enhanced enzymatic activity was discovered. Molecular dynamics simulations of mutant ILRAC and wild-type ASNase were used to further investigate the structural stability. Homology modeling (in SWISS-MODEL), molecular docking and molecular dynamics modeling (in AMBER 18) were used to establish the relationship between changes in protein conformation and an increase in enzyme activity due to the modified residues. The mutant ILRAC K_m_ value decreased to 1.45 mM from 2.33 mM in the wild type, while the mutant ILRAC k_cat_ value increased to 778.87 min^−1^ from 197.95 min^−1^ in the wild type [278].

Rational modification of L-ASNase from *Bacillus licheniformis* (BliA) was carried out to obtain an enzyme with better catalytic properties and half-life. This L-ASNase was chosen because natural BliA lacks glutaminase activity. MSA of L-ASNase protein sequences from *E. coli* (EcA), *Erwinia carotovora* (ErA), *Pseudomonas sp.* (PGA), *P. furiosus* (PfA) and *B. licheniformis* using CLUSTAL 2.1 showed that BliA is very similar to PfA compared to EcA, ErA and PGA. BliA was altered by site-directed mutagenesis using the overlap extension approach. Based on the sequence alignment of previously mutagenized L-ASNases, D103 and G238, four alternative sites, D103, G238, E232 and Q112, were selected to improve hydrophobicity at the dimer interface and increase electrostatic potential. A methodical approach to enzyme engineering resulted in four mutants: G238N, E232A, D103V and Q112H. The mutant D103V enzyme had a specific activity of 597.7 IU/mg, which was higher than that of the native (rBliA) enzyme (407.65 IU/mg). In addition, the D103V mutant BliA was superior to natural BliA at the optimal temperature and in vitro half-life, showing resistance to higher temperatures and a 3-fold longer half-life. Kinetic experiments showed that the D103V mutant L-ASNase has a higher substrate affinity, with a K_m_ of 0.42 mM and a Vmax of 2778.9 µmol min^−1^. Thus, the change from D to V at position 103 is responsible for the improved catalytic properties of BliA [279].

### 4.4. Modifications to Increase Thermostability

**Figure 10:**
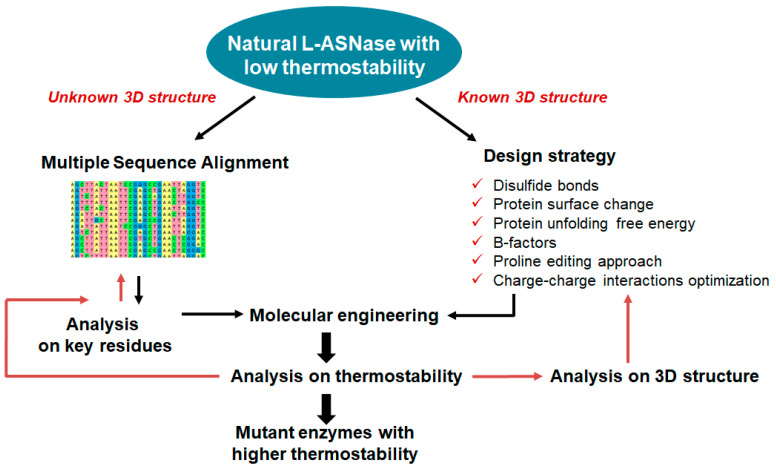
Rational design strategies for improving the thermostability of L-ASNase. Rational design can be used to identify structural flaws in proteins and modify them to increase thermostability: (1) in the case of unknown 3D structure (left), information was obtained by sequence analysis with construct MSA. The most abundant amino acid at each position in a protein sequence often significantly affects stability. Then, 3D modeling, protein stability prediction algorithms and others are used to analyze key residues and predicted residue contacts; (2) when the 3D structure of a protein is known (right), it can be examined to find regions that are prone to unfolding or destabilization at high temperatures. The overall thermostability of the protein can be improved by strengthening these regions using molecular dynamics, strategies based on disulfide bonds, protein surface charge optimization, homologous comparison strategies, design strategy using proline effect analysis and genetic engineering.

The improvement in the thermostability of L-ASNase is crucial for the extension of the range of applications of L-ASNase in the preprocessing of food [280]. The thermal stability of L-ASNase can effectively enhance its ability to reduce the amount of acrylamide [281]. To prevent the formation of acrylamide, different forms of L-ASNase can be used in fried potato products [282]. Random mutagenesis and B-factor screening are often used to improve protein thermostability at the expense of screening thousands of clones (see Figure 10). The computational design of stability-enhancing mutations is a reliable alternative to screening-based approaches, but reliable methods need to be developed to improve prediction accuracy [283]. Several studies have used rational design to develop L-ASNases with increased thermal stability.

Jiao et al. used heterologic expression in *E. coli* BL21(DE3) and consensus design in the Consensus Finder web server to mutate cysteine residues to improve the thermostability of L-ASNase from *Acinetobacter soli* Y-3 (AsA). AcA has low thermal stability due to unstable cysteine residues and mismatched intermolecular disulfide bonds. Cys8 and Cys283 of *Acinetobacter soli* L-ASNase (AsA) were screened for non-conserved cysteine residues with readily oxidizable free sulfhydryl groups, and mutagenesis was performed. A C8Y/C283Q mutant with dramatically increased thermal stability was produced by saturation mutagenesis and combinatorial mutation, with a half-life of 361.6 min at 40 °C, more than 34 times longer than that of the wild type. Its melting point (Tm) is 62.3 degrees Celsius, which is 7.1 degrees Celsius higher than that of the wild type. The evolution of novel hydrogen bonds of Gln283 and the aromatic interaction of Tyr8 with neighboring residues were identified using molecular dynamics modeling in GROMACS and structural analysis in the SWISS-MODEL online server. Thus, the additional hydrogen bonds and conjugation forces induced by the mutation contribute significantly to the heat stability of the mutants. The acrylamide concentration of potato chips was efficiently reduced by 86.50% after treatment with the C8Y/C283Q mutant, which was much higher than the 59.05% reduction after treatment with wild-type AsA [281].

The thermal stability of BlAsnase from *Bacillus licheniformis* was improved using rational design methods. PoPMuSiC was used to predict important possible mutation hotspots and appropriate replacements to increase the thermal stability of the L-ASNase protein. Ranking was used to select amino acid regions with low free energy values based on folding free energy calculations. Furthermore, their conservation was checked by sequence comparison, and the spatial position of the corresponding amino acids in the protein structure was observed using PyMOL to avoid the negative effect of mutations at specific sites on the enzymatic activity of BlAsnase, thus eliminating some inappropriate mutation sites. AMBER 18 software was used to perform all-atom molecular dynamics simulations on dimeric compounds. The structure is rigid, there are hydrophobic contacts, and the electrostatic potential is favorable. The results showed that the D172 W/E207A double mutant had a much stiffer and more stable protein structure than the wild-type BlAsnase. This mutant outperformed wild-type BlAsnase in terms of thermal stability, with a 65.8-fold longer half-life at 55 °C and a 5 °C higher optimum reaction temperature and melting point [261].

In another study, a stable recombinant *R. miehei* L-ASNase was rationally designed using computer design software, conservativeness analysis and functional region evaluation. Using the SWISS-MODEL service and the crystal structure of guinea pig L-ASNase (PDB code: 4R8L) as a homology modeling template, a 3D model structure was generated based on the amino acid sequence of *R. miehei* L-ASNase. As the flexible amino acid residues of an enzyme are typically modified to increase thermal stability, flexible amino acid residues outside the active site were modeled using FoldX. The effect of amino acid changes on G was modeled using FoldX 5. In order of G from smallest to largest, mutants S302M, S302L, D161M, D184M, S302I, E217M, E217P and S302V were created. The best mutants were then overexpressed intracellularly in *B. subtilis* 168. The S302I and S302M mutants thrived at 50 °C. The residual activity of the WT enzyme was only approximately 10% when the mutant and wild-type enzymes were incubated at 45 °C for 35 h. However, the residual activity of S302I and S302M was more than 50%. A combination *B. subtilis* 168-pMA5-A344E/S302I mutant was produced that had high enzymatic activity and good stability [16].

### 4.5. Modifications to Decrease Immunogenicity

Protein immunogenicity can be reduced by altering regions within the target protein that are likely to be recognized as B- or T-cell epitopes by the adaptive immune system. However, the identification and removal of B-cell epitopes is extremely challenging due to their conformational nature. This is further complicated by a poor understanding of the naive antibody repertoire and how it changes in different human populations. Mutagenesis has been successfully used to reduce the immunogenicity of humanized and chimeric antibodies by removing T-cell epitopes. However, although these proteins have only a few relatively short potentially immunogenic regions, heterologous enzymes that have not been immunologically tolerated are likely to have many T-cell epitopes. Extensive mutagenesis of the polypeptide sequence, while retaining the original catalytic activity, would therefore be required to remove these epitopes. Furthermore, in addition to the active site residues, a network of amino acids present throughout the protein influences enzyme catalysis [284].

Computational techniques to address protein immunogenicity issues are gaining popularity. Deimmunogenization of therapeutic proteins by altering T-cell or B-cell epitopes using bioinformatic tools and site-directed mutagenesis has been shown to be an effective technique for creating safer biopharmaceuticals. Based on these concepts and the high immunogenicity of *E. coli* L-ASNase in 2019, Belén et al. created an in silico chimera of L-ASNase by replacing epitope peptides in the *E. coli* enzyme variant with peptides from human serum albumin and even demonstrated proof of concept that the engineered variant is recombinantly expressed in *E. coli* [285].

#### 4.5.1. T-Cell Epitope Prediction and Allergenicity Prediction

A humanized chimeric enzyme with reduced immunogenic potential of *E. coli* L-ASNase was generated in silico by rational design. For this purpose, immunogenic *E. coli* L-ASNase epitopes (PDB: 3ECA) were discovered and substituted by less immunogenic *Homo sapiens* L-ASNase (PDB: 400H). PyMOL software (https://pymol.org/2/) was used to model the structures, and the SWISS-MODEL service was used to model the chimeric enzyme. Protein-ligand docking predicted the presence of asparaginase enzymatic activity in a humanized chimeric enzyme with four subunits comparable to the template structure. The NetMHCII 4.0 server was used to predict T-cell epitopes. AllerTOP v. 2.0 was used to predict allergenicity. This tool was used to process each T-cell epitope predicted to be immunogenic for the HLA-DRB1*04:01 and HLA-DRB1*07:01 alleles. The program analyses the peptide sequence and provides a ‘probable allergen’ or ‘probable nonallergen’ result. Although the difference is not significant, the human enzyme has proven to be a good alternative, as it is less immunogenic than the bacterial enzyme. The current study enabled the development of a stable chimeric enzyme for the treatment of acute lymphoblastic leukemia, identical to the commercially tested native form [286].

A bioinformatics-based technique was used to analyze and select in silico L-ASNases from Streptomyces for the treatment of ALL. Active site prediction tools and molecular docking were used to select an enzyme with high affinity for L-asparagine, and antigenicity and protein structure prediction tools were used to select an enzyme with lower immunogenicity. Identification and selection of homologous L-ASNases was performed using the amino acid sequences of EcAII (ID P00805) and *Streptomyces coelicolor* II L-ASNase (ScAII; ID Q9K4F5) as seeds to yield putative L-ASNases from Streptomyces. The ANTIGENpro server was used to calculate the probability of antigenicity of each ASNase. The amino acid sequence of each candidate L-ASNase was screened for T-cell epitopes using the Immune Epitope Database server to search for high affinity binding sites. *S. coelicolor* (Q9K4F5), *S. scabrisporus* (WP_078980718.1) and *S. albireticuli* (ARZ68596.1) were selected as potential enzymes with reduced allele coverage, epitope density (ED) and antigenicity probability. For these enzymes, homology modeling (I-TASSER) and molecular docking (AutoDock) were performed. L-ASNases from the PF06089.11 family have lower EDs and fewer epitope clusters across the sequence than enzymes from the PF00710.11 family [272].

#### 4.5.2. Prediction of the Toxicity of the Protein

ToxinPred software (http://crdd.osdd.net/raghava/toxinpred/) predicts peptide toxicity using a machine learning technique and a quantitative matrix based on various features of the peptides. ToxinPred’s protein scanning module was used to discover a particularly hazardous region in L-ASNase [287]. Toxin red, PoPMuSiC, KoBamin and the I-TASSER server were used in a study to generate a novel structure for L-ASNase from *E. coli* in silico that reduced toxicity while increasing stability and half-life. Six protein sequences were obtained, with the mutant-6 sequence proving to be the best because of four structural changes: L23G, K129L, S263C and R291F. The newly designed protein was nontoxic, had a half-life 25 h longer and was 600 kcal/mol more stable than the wild type, with no significant changes in the protein’s secondary or tertiary structure, antigenicity or allergenicity [260].

Conjugation of biocompatible chemical polymers, such as PEG, has already been used to increase half-life and hide antibody epitopes on therapeutic protein surfaces. One drawback of PEG application is a poorly controlled heterogeneous mixture of PEG-conjugated isomers and immunogenicity, with anti-PEG antibodies present in up to 70% of the general population. Recently, protein resurfacing was used to avoid the effects of pre-existing anti-L-ASNase antibodies by creating proteins with extensive surface residue modifications. *E. coli* L-ASNase mutants with up to 58 changes were generated, resulting in direct modification of 35% of surface residues. dTERMen, pyRosetta and I-TASSER were used to design resurfaced L-ASNase variants. These methods were used to introduce mutations into the wild-type *E. coli* L-ASNase reference sequence (PDB ID: 3ECA) while maintaining the overall expected structure, maximizing the influence on surface-exposed amino acids and minimizing the risk of affecting protein folding or enzymatic function. Binding of resurfaced L-ASNases to human polyclonal anti-ASN antibodies was dramatically reduced, with a negative association between binding and mutational distance from the native protein. Reduced anti-L-ASNase antibody-binding was associated with reduced hypersensitivity in an in vivo mouse model [288,289].

To overcome the disadvantages of L-ASNase and PEG-ASNase, Zhang et al. developed a genetic construct called L-ASNase-ELP (L-ASNase linked to an elastin-like polypeptide, ELP). AlphaFold2 predicted the structure of the chimera. Long after injection, the temperature sensitivity of the L-ASNase-ELP chimera resulted in the formation of an in situ depot with sustained release of the enzyme and zero-order release kinetics. In vitro and in vivo studies also showed that compared to L-ASNase and PEG-ASNase, L-ASNase-ELP had higher activity retention, superior stability, longer half-life, lower immunogenicity, lower toxicity and higher potency in vivo and in vitro [290].

In this way, L-ASNases with improved properties have been obtained by means of rational design, directed evolution and heterologous expression (see Table 2).

## 5. Conclusions

The evolution of host strains has been influenced by changes at many different levels, including site-directed mutagenesis, promoters, transcription factors, gene copy number, codons, chaperones, leader peptides and structural levels such as glycosylation and enzyme folding [292].

Through improving the physicochemical properties of engineered proteins, selecting the best cell line, and optimizing culture media and expression conditions, it is possible to produce recombinant proteins more efficiently and with higher yields while reducing the number of time-consuming and costly steps involved. Experimental methods for screening potential drug compounds are still very expensive and take a long time. A more efficient method is to pretest a large library of small molecules in silico and then select a small group for experimental validation. In addition, the availability of data on the three-dimensional structure of enzymes and substrates makes it possible to analyze their interaction energy and other parameters [277]. Computational approaches are often used in pharmaceutical biotechnology research to create new types of protein therapeutics with improved performance [293].

Today, the application of modern computational tools and directed evolution approaches can alter the evolution of enzymes. The combination of directed evolution with rational and semi-rational strategies, including molecular dynamics and quantum mechanics/molecular mechanics simulations, is expected to lead to the development of enzymes with improved biotechnological properties in the coming years. It is a promising approach to identify targets for protein engineering and reduce library screening efforts [294]. Therefore, suitable heterologous expression systems with tolerance to high enzyme expression levels should be developed for enzymes. For example, directed evolution combined with hybrid techniques has led to functional heterologous expression and activity, adaptation to non-natural habitats and the creation of laccase chimeras with mixed characteristics [295]. Rational design and heterologous expression in *E. coli* were used to obtain a new version of the thrombolytic drug, Reteplase [296].

Recently, the thermostability of L-ASNase from *R. miehei* and *Acinetobacter soli* has been improved via heterologous expression and rational design [16,281]. This approach also resulted in L-ASNase with improved stability, half-life, reduced immunogenicity, in vitro and in vivo anti-cancer activity. In addition, this L-ASNase was more active than the native or PEG-conjugated enzyme [290].

Therefore, in silico modeling has recently emerged as a critical technique for identifying defects at the molecular level, and the data collected can be used to modify the native L-ASNase structure. Because this method avoids costly, labor-intensive and time-consuming experimental designs, it may become more common in the future [20]. Rational design can provide insight into the structure-function relationship of L-ASNase. By studying the effects of specific mutations on the activity and stability of the enzyme, researchers can gain a better understanding of the structure and function of the protein. This knowledge can be used to guide future protein engineering efforts and improve the efficiency of the rational design process.

## Figures and Tables

**Table 1:** Methods of directed evolution.

Random Mutagenesis		References
Error-prone PCR (epPCR)	A modification of the standard PCR method that is designed to alter and increase the natural error rate of the polymerase. Random mutations may be introduced into the DNA.	[204]
Sequence saturation mutagenesis (SeSaM)	Can be used to target one type of nucleotide in the selected sequence, and each type of nucleotide can be exchanged in a controlled manner. Completely independent of DNA polymerase mutation biases.	[205]
Cassette mutagenesis	The modification of a protein sequence at the level of the DNA by the replacement of a section of genetic information with an alternative sequence, usually provided by a synthetic duplex of DNA. Single or multiple amino acid changes to the protein sequence, as well as the insertion or deletion of sequences from the protein structure, can be achieved using this technique.	[206]
Site-saturation mutagenesis (SSM)	A strategy for the generation of high quality variant gene libraries of a defined size. Variation is introduced by creating a precise series of amino acid substitutions in the encoded protein through the incorporation of degenerate base combinations at specific codon positions.	[207]
Nondegenerate saturation mutagenesis	Contiguous (ProxiMAX) and noncontiguous (MAX) randomized codon generation techniques to create well-defined, diverse gene libraries in conjunction with other fully nondegenerate strategies.	[208]
The Darwin Assembly	Multiple site-saturation mutagenesis method capable of targeting up to 19 noncontiguous residues with less than 0.25% wild-type contamination. The assembly strategy has been designed with automation in mind, accelerating library construction and enabling the generation of highly complex libraries with minimal hands-on time and low potential for human error.	[209]
Recombination mutagenesis		
DNA shuffling	Method for reassembling genes from their random DNA fragments, resulting in in vitro homologous recombination. Insertions and deletions result in nucleotide addition or removal.	[210]
Staggered extension protocol (StEP)	A modified PCR that generates staggered DNA fragments and promotes crossover events along the full length of the template sequence(s) using greatly shortened annealing and extension steps. Can be performed in a single tube and does not require DNA fragmentation.	[211]
Incremental truncation for the creation of hybrid enzymes (ITCHY)	Technique for the random recombination of two genes. The main advantage of ITCHY is that it does not require the two genes to share any sequence similarity.	[212]
Random chimeragenesis on transient templates (RACHITT)	Generates libraries with an average of 12 crossovers per gene in a single round of gene family shuffling. Generates chimeric genes by alignment of parental gene ‘donor’ fragments to a full-length DNA template	[213]
Rational mutagenesis		
Site-directed mutagenesis	Specific point mutations can be introduced into plasmids by the use of primers (with the desired mutation) in a PCR protocol that will amplify the entire plasmid template.	[206,214]
Site-directed mutagenesis method mediated by Cas9	Site-directed mutagenesis in a modern form. CRISPR-Cas9 systems can be programmed to target any locus in the genome and to carry out targeted CRISPR-mediated directed evolution.	[214,215]
Computer-assisted recombination (CompassR) strategy	Enables the effective and efficient recombination of identified beneficial substitutions to produce active enzymes with improved performance. By analyzing the relative free energy of folding, which has been used as a measure of protein stability and to assess the relationship between stability and function in several enzymes, the CompassR strategy guides the recombination of beneficial substitutions.	[216]

**Table 2:** Examples of the improvement of L-ASNase properties using directed evolution and rational design approaches.

Source	Host	Method	Improvement	Reference
Directed evolution				
*Bacillus megaterium* H-1	*E. coli* BL21(DE3)	epPCR, DNA shuffling	Catalytic activity	[217]
Guinea pig, human	*E. coli* BW5Δ strain	epPCR, DNA shuffling, CAST mutagenesis	Catalytic activity	[218]
*Erwinia carotovora* and *E. chrysanthemi*	*E. coli* BL21(DE3)pLysS	StEP, site-saturation mutagenesis	Increased thermostability	[219]
*E. chrysanthemi* and *Erwinia carotovora*	*E. coli* BL21(DE3)pLysS	StEP	Decreased glutaminase activity	[220]
Human	*E. coli* strain JC1(DE3), C41(DE3)	Site-saturation mutagenesis, epPCR, high-throughput screening	Increased catalytic activity	[221]
Rational design				
Halophilic bacteria	in silico design	Detection of rare codon cluster, molecular modeling and model evaluation, molecular docking, structural analysis of rare codon.	Increased expression	[269]
*E. coli*	in silico design	Genetic algorithm, molecular dynamics	Enhanced anticancer activity, decreased glutaminase activity	[66]
*E. chrysantemi*	*E. coli* BL21(DE3) C41	Codon optimization, MSA, molecular dynamics	Decreased glutaminase activity	[109,117]
*E. coli* L-ASNase II	-	Saturation mutagenesis, molecular dynamics, enzymological screening	Decreased glutaminase activity	[103]
*E. coli* L-ASNase II	in silico redesign	Molecular dynamics, quantum mechanics molecular dynamics, molecular docking	Decreased glutaminase activity	[273]
*E. coli* L-ASNase II	*E. coli* CU1783	Site-directed mutagenesis, MSA, molecular dynamics	Decreased glutaminase activity	[274]
*Pectobacterium carotovorum*	in silico	Molecular dynamics, molecular docking	Decreased glutaminase activity	[275]
*Bacillus licheniformis*	*E. coli* BL21 (DE3)	MSA, site-directed mutagenesis	Increased catalytic activity and half-life	[279]
*E. coli* and *Erwinia* species	in silico design	Phylogenetic analysis, homology modeling, active site prediction, molecular docking	Increased catalytic activity	[277]
*E. coli* L-ASNase II	in silico design	Phylogenetic analysis, homology modeling, active site prediction, molecular docking	Increased catalytic activity and low K_m_	[277]
*Acinetobacter soli* Y-3	*E. coli* BL21 (DE3)	Consensus design, homology modeling, molecular dynamics	Improved thermostability	[281]
*Bacillus licheniformis*	*E. coli* BL21 (DE3)	Prediction important possible mutation hotspots, MSA, site-directed saturation mutagenesis, molecular dynamics	Improved thermostability	[261]
*Rhizomucor miehei*	*B. subtilis* 168	Homology modeling, molecular dynamics, site-directed mutagenesis	Improved thermostability	[16]
*Homo sapiens*	in silico design chimeric humanized L-ASNase	T-cell epitope prediction and epitope density determination, prediction of allergenic epitopes, homology modeling, MSA, machine learning, molecular docking	Decreased immunogenicity	[286]
*Streptomyces* species	in silico search	MSA, phylogenetic analyses, homology modeling, molecular docking, antigenicity prediction, HLA class II binding prediction	Decreased immunogenicity	[272]
*E. coli*	in silico redesign	MSA, predicting protein stability, homology modeling, protein toxicity analysis, predicting antigens and allergens, prediction of B-cell epitopes	Reduced toxicity, increasing stability and half-life	[260]
*E. coli* WT	in silico redesign	MSA, homology modeling, anti-His-tag ELISA, de novo design, redesign of mCherry Surface	Decreased immunogenicity	[288,289]
In silico design	*E. coli* Rosetta (DE3)	Machine learning	Decreased immunogenicity, low toxicity	[290]
Semi-rational design				
*Bacillus licheniformis*	*E. coli* BL21 (DE3)	Coevolution analysis, screening of mutant libraries, saturation mutagenesis, homology modeling, molecular dynamics, molecular docking	Increased catalytic activity	[278]
*Mycobacterium gordonae*	*E. coli* BL21 (DE3)	Site-directed saturation and combinatorial mutagenesis, high-throughput screening, consensus design, homology modeling, molecular dynamics	Improved thermostability	[291]

## Data Availability

The data presented in this review are available on request from corresponding author.
